# Efficacy and cost-effectiveness of a digital guided self-management intervention to support transition from intensive care to community care in anorexia nervosa (TRIANGLE): pragmatic multicentre randomised controlled trial and economic evaluation

**DOI:** 10.1016/j.eclinm.2024.102645

**Published:** 2024-05-27

**Authors:** Valentina Cardi, Katie Rowlands, Suman Ambwani, Jodie Lord, Danielle Clark-Bryan, David McDaid, Ulrike Schmidt, Pamela Macdonald, Jon Arcelus, Sabine Landau, Janet Treasure

**Affiliations:** a Centre for Research in Eating and Weight Disorders, Institute of Psychiatry, Psychology and Neuroscience, King's College London, London, UK; b Department of General Psychology, University of Padova, Padova, Italy; c DIS Study Abroad in Scandinavia, Copenhagen, Denmark; d Department of Biostatistics and Health Informatics, Institute of Psychiatry, Psychology and Neuroscience, King's College London, London, UK; e Care Policy and Evaluation Centre, Department of Health Policy, London School of Economics and Political Science, London, UK; f South London and Maudsley NHS Foundation Trust, London, UK; g Institute of Mental Health, University of Nottingham, Jubilee Campus, Triumph Road, Nottingham, NG7 2TU, UK; h Bellvitge Biomedical Research Institute (IDIBELL), Hospitalet del Llobregat, Barcelona, Spain

**Keywords:** Anorexia nervosa, Carers, Digital, Groups, Psychoeducation, Recovery, Self-management

## Abstract

**Background:**

There is uncertainty regarding how best to support patients with anorexia nervosa following inpatient or day care treatment. This study evaluated the impact of augmenting intensive treatment with a digital, guided, self-management intervention (ECHOMANTRA) for patients with anorexia nervosa and their carers.

**Methods:**

In this pragmatic multicentre randomised controlled trial and economic evaluation, patients with a diagnosis of anorexia nervosa or atypical anorexia nervosa, aged 16+ and attending one of the 31 inpatient or day-patient services in the UK were randomised with one of their carers to receive ECHOMANTRA plus treatment as usual (TAU), or TAU alone. ECHOMANTRA was hosted on a digital platform and included a workbook, recovery-oriented video-clips and online facilitated groups (patients only, carers only, joint patient-carer). Participants were randomised on a 1:1 ratio using a minimisation algorithm to stratify by site (N = 31) and severity (defined by BMI <15 and ≥ 15 kg/m^2^ at baseline). The primary outcome was patient depression, anxiety, and stress at 12 months. Primary and secondary outcomes were compared between trial arms on an intention-to-treat basis (ITT). This trial is registered with the ISRSTN registry, ISRCTN14644379.

**Findings:**

Between July 01, 2017 and July 20, 2020, 371 patient-carer dyads were enrolled and randomly assigned to ECHOMANTRA + TAU (N = 185) or TAU alone (N = 186). There were no significant differences between trial arms with regards to the primary outcome (completed by N = 143 patients in the TAU group, Mean = 61.7, SD = 29.4 and N = 109 patients in the ECHOMANTRA + TAU group, Mean = 58.3, SD = 26.9; estimated mean difference 0.48 points; 95% CI −5.36 to 6.33; *p* = 0.87). Differences on secondary outcomes were small and non-significant (standardised effect size estimates ≤0.25). Five patients died (2 from suicide and 3 from physical complications) over the course of the trial, and this was unrelated to their participation in the study.

**Interpretation:**

ECHOMANTRA added to TAU was not superior to TAU alone in reducing patient depression, anxiety, and stress symptoms. This may be explained by limited engagement with the intervention materials and changes in usual care practices since the beginning of the trial.

**Funding:**

10.13039/501100000272National Institute for Health Research (NIHR), under its 10.13039/501100000664Health Technology Assessment Programme (HTA) Programme (Grant Reference Number 14/68/09). 10.13039/100019418NIHR Maudsley Biomedical Research Centre (BRC), 10.13039/100009362South London and Maudsley NHS Foundation Trust and 10.13039/100013376Institute of Psychiatry, Psychology and Neuroscience, and King’s College London. 10.13039/501100023232NIHR Applied Research Collaboration South London (NIHR ARC South London) at 10.13039/100010872King's College Hospital NHS Foundation Trust.

**Research in context**

**Evidence before this study**

Admissions and readmission for anorexia nervosa have been steadily increasing for over 10 years. One reason is the difficulty to sustain patients and their families through treatment transitions. In 2020, we undertook a systematic review of the literature on transition support for anorexia nervosa. We searched the databases EMBASE (1974–2020), MEDLINE (1946–2020), PsycINFO (1806–2020) and Web of Science (1900–2020) for studies describing interventions to bridge the gap between hospitalization and post-hospitalisation for adult patients with anorexia nervosa and which reported clinical outcomes and drop-out rates for a minimum of two months following discharge from intensive treatment. Fourteen papers were included in the analysis. Drop-out rates ranged from 10% to 42% and small to medium sized improvements in weight, eating and general psychopathology were found. However, there was a lack of randomised controlled trials and samples were small.

**Added value of this study**

We conducted the largest randomized controlled trial of a transition intervention for anorexia nervosa, to date. The intervention materials could be accessed through an online platform and consisted of resources developed with people with lived experience of the illness. The use of the intervention materials was suboptimal, with only 20% of patients and families reaching the adherence criterion. The feedback from this study suggests that ambivalence towards change and the heavy burden posed by the illness and its treatment need to be addressed in more personalised forms of aftercare interventions.

**Implications of all the available evidence**

Digital interventions based on lived experience hold potential. Patients with anorexia nervosa and their carers are often exhausted by treatment efforts and might struggle to sustain motivation to change through the recovery journey. The use of digital interventions in anorexia nervosa might need greater personalisation, regular monitoring and feedback, and therapeutic guidance for patient benefit.

## Introduction

There is a high level of uncertainty about treatment of patients with anorexia nervosa. An umbrella review concluded that there is little evidence to suggest that any form of psychotherapy produces better outcomes, with the possible exception of interventions involving families.^1^ Approximately 20% of adult patients with anorexia nervosa develop high medical or psychological risks and require inpatient or day patient care.^2^ There has been a call for more research in this area of management as the procedures followed vary^3^ and the overall outcomes are poor.^4^ Similarly, there is a high level of uncertainty around the best protocols for supporting patients after inpatient care. These “aftercare” interventions were reviewed recently following the PRISMA guidelines, and include pharmacological treatments and forms of individual, family and digital support, which tend to produce only small to moderate benefits.^5^^,^^6^

We have developed two work streams to implement “aftercare” interventions for patients with anorexia nervosa. Both followed the procedures for the development of complex interventions described by the UK Medical Research Council.^7^ The first stream, “Experienced Carers Helping Others” (ECHO), included a digital, telephone-guided intervention for carers. This produced a small to moderate reduction in patient time spent in hospital and in patient and carer distress.^8^^,^^9^ The second, “iMANTRA” was a digital, guided, aftercare adaptation of the Maudsley Model of Anorexia Nervosa Treatment for Adults (MANTRA) which, in a feasibility study, improved patient body mass index and depression outcomes at 12 months.^10^ Additionally in the “Self-Help Aid and Recovery Guide for Eating Disorders” (SHARED) study, lived experience recovery narratives were added to form “Recovery MANTRA,” which, when given as a form of preparatory care on the waiting list for standard therapy, produced a small reduction in anxiety, increased confidence to change, and improved therapeutic alliance with the outpatient therapist.^11^

The current study, “Transition care in anorexia nervosa through guidance online from peer and carer expertise” (TRIANGLE) included materials from the ECHO intervention, primarily for carers, and materials from Recovery MANTRA, primarily for patients.^12^ The protocol name, “TRIANGLE,” was chosen to signify the involvement of patients, carers, and professionals in the treatment of anorexia nervosa. This combination has shown benefits in a proof-of-concept study.^13^ The primary objective of this trial was to examine whether patient and carer dyads randomised to receive the ECHOMANTRA intervention in addition to Treatment as Usual (TAU) during intensive care would demonstrate a greater reduction in symptoms of depression, anxiety, and stress 12 months post-randomisation compared to patients who were randomised to receive TAU alone. This outcome was decided based on feedback from people with lived experience of the illness and on the evidence that affective symptoms are core predictors of the course of eating disorders.^14^^,^^15^ In addition, patients identify mental health care as a crucial unmet need.^16^

## Methods

### Study design

TRIANGLE was a pragmatic, two arm, multicentre, parallel group, randomised controlled trial and economic evaluation of a digital self-management intervention for patients with anorexia nervosa attending intensive care (day or inpatient care) and their carers. The trial was approved by the London–Camberwell St Giles Research Ethics Committee (Reference: 16/LO/1377) in the UK. The protocol was published before recruitment began.^12^ Changes to the protocol occurred over the course of the trial, which were discussed and agreed upon by the Trial Steering Committee (TSC) and the Data Monitoring Committee (DMC). These changes were approved by the London–Camberwell St Giles Research Ethics Committee (Reference: 16/LO/1377) and are described in detail in Supplementary Materials 1. The main amendments to the published protocol included: 1) extension of time window to participate in the trial (from four weeks post-admission to four months post-discharge); 2) broadening of patient inclusion criteria (age lowered to 16; attendance of intensive treatment for a minimum of three days/week; BMI greater than 18.5 kg/m^2^); 3) replacement of video call sessions with patients and carers joint online groups; 4) change to the outcome measure for number of days spent in hospital at 12 months, from Hospital Episode Statistics to the self-report Client Service Receipt Inventory. The main motivations to make these amendments were to increase the chances that interested participants could take part (changes n. 1, 2), to reduce the burden of participation (change n. 3) and to deliver on the assessment of days spent in hospital as a secondary outcome (change n. 4).

All participants received the care that would normally be delivered at each participating centre (N = 31 inpatient or daycare services in the UK) referred to as Treatment as Usual (TAU). Nineteen of the 31 centres participating in TRIANGLE were accredited by the Quality network for Eating Disorders (QED Royal College of Psychiatrists). The network provides a rigorous review of services, including adherence to care standards pertaining to the involvement of carers and post-discharge planning. Patient/carer dyads were randomly allocated to TAU alone, or the ECHOMANTRA intervention plus TAU (TAU + ECHOMANTRA). Those allocated to the ECHOMANTRA intervention plus TAU arm could access the ECHOMANTRA materials immediately after randomisation. Dyads were followed up for 18 months after randomisation.

The authors adhered to the appropriate EQUATOR reporting guidelines (i.e., CHEERS and CONSORT).

### Participants

Patient/carer dyads were eligible to join the study if the patient had a diagnosis of anorexia nervosa or atypical anorexia nervosa (Diagnostic and Statistical Manual of Mental Disorders, Fifth Edition: DSM-5), was aged 16 (or over), was admitted to an inpatient/or day patient unit for a minimum of three days/week, and if both had access to an electronic device (e.g., mobile phone, computer, laptop, tablet) and the Internet (in order to use the study website). Nominated carers were non-professional sources of social support (i.e., family member, partner or friend). Patients were ineligible if they had additional severe mental or chronic physical illness needing specific treatment (e.g., psychosis, diabetes mellitus, cystic fibrosis etc.) or if the patient was pregnant. Patients and carers were ineligible for inclusion if they had insufficient knowledge of English or had received previous treatments involving the ECHOMANTRA intervention materials.

Written informed consent was obtained from both the patient and their carer before randomisation. Subsequently, the Research Assistants sent separate emails to the patient and carer with individual login details to the study website (created by Mindwave; http://mindwaveventures.com), where they could complete the baseline questionnaires, as well as the follow-up measures. Study champions and/or the trial Research Assistants provided guidance if needed via email/telephone or in-person. Participants completed self-report questionnaires through the study's website.

### Randomisation and masking

After completing the baseline assessment, patient/carer dyads were randomised to either TAU + ECHOMANTRA or TAU alone on a 1:1 ratio using a minimisation algorithm to stratify by site and severity (defined by BMI <15 and ≥ 15 kg/m^2^ at baseline). Randomisation was delivered by the King's Clinical Trials Unit (CTU). Neither the patient nor carer were blind to the treatment arm when completing questionnaires post-randomisation. Clinicians at the inpatient or day patient centre were kept blind. The senior statistician remained partially blind (knowing only coded trial arm's membership) until as late as possible into primary analyses being conducted. The junior statistician was unblinded after database lock.

### Procedures

The trial was coordinated at the Institute of Psychiatry, Psychology and Neuroscience, King's College London, managed by a Trial Management Group (TMG), which met monthly, and it was overseen by a Trial Steering Committee (TSC) and a Data Monitoring Committee (DMC), which met seven times in total over the lifetime of the trial (5 years).

Liaison with each site was through study champions where possible (these included local Principal Investigators, Research Nurses/Assistants or other key clinical staff from each Trust), or staff from the Clinical Research Network (these were Clinical Studies Officers).

#### Treatment as usual (TAU) at the participating centres

TAU followed the guidelines of the UK Quality network for Eating Disorders (QED) of the Royal College of Psychiatrists for inpatient and outpatient care. These include developing a personalised care plan with patients and carers, the delivery of evidence-based therapeutic interventions (based on the NICE guidelines), undertaking structured activities, such as education and volunteering, regular medical reviews, and active involvement of carers. It is possible that some details of care varied due to local commissioning and design.

#### ECHOMANTRA: materials and definition of adherence

Participants randomised to the ECHOMANTRA intervention arm received access to the study's website immediately after randomisation. The study's website hosted a patient and a carer's workbook and a library of videoclips featuring people with lived experience of eating disorders or mental health professionals working in the field. The workbooks and the videos discussed information about predisposing and maintaining factors of the illness (e.g., the impact of poor nutrition on the brain and the body, interpersonal reactions to the eating disorder symptoms, cognitive rigidity and emotion regulation, self-care) and provided tips for behaviour change. These same topics were discussed in the context of eight patient-only, carer-only or joint patient-carer groups. In particular, the patient groups' themes included the impact of the eating disorder on the brain and the body, the characteristics of the eating disorder, the importance of self-compassion, the impact of the disorder on social life, the animal models of carers' reactions to the illness, understanding and managing emotions, making changes and planning for transition, and managing mealtimes and preventing relapse. The carer groups included a discussion of risk factors, strengths and resources, carers' resilience, common emotional reactions to the eating disorder, practising compassion and compassionate communication, planning and facilitating transition and meal support. The joint carer-patient groups focused on social networks, behaviour change, personal growth and recovery identity, and nutritional support. The online groups occurred weekly. They were moderated and facilitated by the study team. Each “group cycle” included eight online meetings. Participants could joint as many groups as desired. The groups were advertised on the trial's website and participants could book their place. Participants were informed about upcoming groups in weekly email notifications and transcripts of the groups were available on the platform to allow for wider access.

Adherence to ECHOMANTRA was defined as both the patient and carer participating in at least four online groups. This was decided based on the acceptability data collected in a previous study on guided self-help in patients with anorexia nervosa.^17^ In that study, patients in the treatment arm were offered some of the self-help materials also used in ECHOMANTRA, in addition to six online chat-based sessions with a mentor. All patients who completed the main outcome assessment at six weeks were able to join at least four of the six online sessions.^17^ In TRIANGLE, participation in an online group was defined as posting at least one message during the group.

### Outcomes

The primary outcome was patients’ depression, anxiety, and stress symptoms (total score on the Depression, Anxiety and Stress Scales-21)^18^ at 12 months post-randomisation. Secondary outcomes for patients included:

• Depression, Anxiety and Stress Scales (DASS-21)^18^ scores at 18 months post-randomisation.
• Body Mass Index (BMI) at 12- and 18 months post-randomisation. This was primarily self-reported, to avoid burden on clinicians.
• Eating disorder psychopathology (EDE-Q)^19^ at 12- and 18 months post-randomisation.
• Work and social adjustment (WSAS)^20^ at 12- and 18 months post-randomisation.
• Importance and ability to change (Motivational Ruler) at 12- and 18 months post-randomisation.
• Social functioning, as reported by carers, via the Strengths and Difficulties Questionnaire (SDQ)^21^ at 12 months post-randomisation.
• Health-related quality of life assessed using the European Quality of Life 5-Dimensions 3-Level Version (EQ-5D-3L)^22^ at 12 months post-randomisation.
• Number of days that patients spent in hospital at 12- and 18 months post-randomisation, as recorded in Hospital Episode Statistics. It was not possible to retrieve these data from the Hospital Episode Statistics (i.e., data containing details about hospital attendance at NHS hospitals in England) as planned. Therefore, this measure was replaced by the self-reported hospital stay data collected in a customised version of the Client Service Receipt Inventory (CSRI),^23^ with reference to the 3 months prior to completion of the baseline questionnaires, and the 3 months prior to the 12 month follow-up post-randomisation. The CSRI was also used to collect all hospital and community-based health service use, as well as information on productivity losses from work and volunteering.

The DASS, BMI and EDE-Q were also collected at intermediate time points (i.e., 3, 6, and 9 months).

#### Economic measures

• Resource utilisation using an adapted version of the CSRI^23^ at baseline and 12 months post-randomisation.
• Health-related quality of life assessed using the EQ-5D-3L^22^ at 12 months post-randomisation.

Secondary outcomes for carers included:

• DASS-21 at 12- and 18 months post-randomisation.
• Skills to cope with the eating disorder symptoms, measured through the Caregiver Skills Assessment scores (CASK)^24^ at 12- and 18 months post-randomisation.

Further details about each measure are provided in Supplementary Materials 2.

### Statistical analyses

The protocol paper details sample size and power calculations.^12^ In total, 380 dyads were estimated as providing 90% power to detect an effect size of Cohen's d = 0.4 for patient's depression, anxiety, and stress symptoms at 12 months post-randomisation, using a two-tailed t-test at a significance level of 5% and allowing for an attrition rate of 30%.

Primary and secondary patient and carer outcomes were compared between trial arms on an intention-to-treat basis (ITT). To estimate the difference in patient DASS-21 at 12 months between the trial arms, a linear mixed model was used in conjunction with multiple imputation (MI) to allow for the adjustment of missing data biases. The use of Multiple imputation (MI) was necessary because a post-randomisation variable (receipt of ECHOMANTRA) predicted missingness (ECHOMANTRA arm only: χ^2^ (1) = 18.9, *p* < 0.001). An additional predictor of missingness was the baseline variable **“**Ever treated under the Mental Health Act followed by a Community Treatment Order?” (MHA/CTO), which was found to predict missingness of DASS-21 at 12 months at the liberal 10% level. The analysis model was a mixed effects model, which included fixed effects for DASS-21 at baseline, treatment allocation and illness severity, and a site-varying random intercept to account for site differences in outcomes. However, the analyses model did not allow for treatment effects to vary by site (no random coefficient of treatment) to avoid over parameterisation in the context of multiple imputation. The imputation model included recruitment sites as fixed effects. Any sites with fewer than five dyads with values on all outcomes were collapsed with the most geographically close neighbour to avoid over-parameterisation. Multivariate imputation by chained equations^25^ with 100 imputations was used and included the following variables in the imputation step: (i) DASS-21 at 12 months, (ii) DASS-21 at baseline, (iii) DASS-21 at 9 months, (iv) DASS-21 at 18 months, (v) treatment allocation (binary), (vi) dummy coded recruitment site (11 categories), (vii) illness severity (binary), (viii) MHA/CTO (binary predictor of missingness) and (ix) a binary indicator of ECHOMANTRA adherence. Inferences were constructed from multiple imputed datasets using Rubin's rules.

Secondary outcome variables were analysed in a similar fashion using linear mixed models with MI where possible (i.e., DASS-21, BMI, EDE-Q, WSAS, importance to change, ability to change, social functioning, CASK, EQ-5D-3L). For the analysis of days spent in hospital for the three months prior to 12 month follow-up, the analysis model consisted of a negative binomial model to account for overdispersion in this count variable and adjusted for site as a fixed effect.

Three sensitivity analyses were carried out for the primary outcome: (i) To assess the effect of treatment receipt (treatment efficacy), as opposed to treatment assignment (treatment effectiveness), the complier average causal effect (CACE) was estimated using two-stage least squares regression. (ii) To ensure that results were not impacted by the presence of participants fulfilling the criteria for atypical anorexia nervosa rather than anorexia nervosa at the time of randomisation, these participants were removed and the primary analysis model re-run. (iii) This trial started prior to the onset of the COVID-19 pandemic and ended after the acute pandemic period. To check for the impact of COVID, all data collected from participants randomised after 11th March 2020 were excluded (this date was suggested by the members of the DMC, based on the announcement of the first lockdown in the UK). All statistical analyses (including multiple imputation) were carried out in Stata 17.^26^

### Economic evaluation

The economic evaluation was performed from both a healthcare system and a broader perspective, including productivity impact on patients from lost employment and volunteering. Data on health service utilisation and productivity losses for the previous three months were collected at baseline and 12 months using the CSRI. Resource use and costs associated with ECHOMANTRA were obtained from project records. Appropriate unit costs were attached to health service use, including specialist inpatient care, as well as outpatient visits and community service contacts. See Supplementary Materials 3 and 4 for details. All patient productivity losses were valued using age-specific mean wage rates linked to occupation. All costs are in 2022 UK pounds and discounting was not applied given the short duration of follow up.

Given the skewed distribution of costs, differences in mean costs were compared between the two patient groups using bias-corrected and accelerated (BCa) bootstrapping 1000 times. The main outcome of interest in the economic analysis was incremental cost per quality adjusted life year (QALY) gained at 12 months. Statistical uncertainty was explored through bootstrapping 1000 randomly resampled pairs of costs and outcomes and cost-effectiveness planes generated. Cost-effectiveness acceptability curves (CEACs) were also generated to show the likelihood of ECHOMANTRA being cost-effective at different willingness to pay levels. A Consolidated Health Economic Evaluation Reporting Standards statement^27^ is provided in Supplementary Materials 5.

### Role of the funding source

The funder of the study had no role in study design, data collection, data analysis, data interpretation, or writing of the report. SL, JT, VC, SA, KR, and JL had access to the dataset. All authors had final responsibility for the decision to submit for publication.

## Results

### Participants flow

**Fig. 1:**
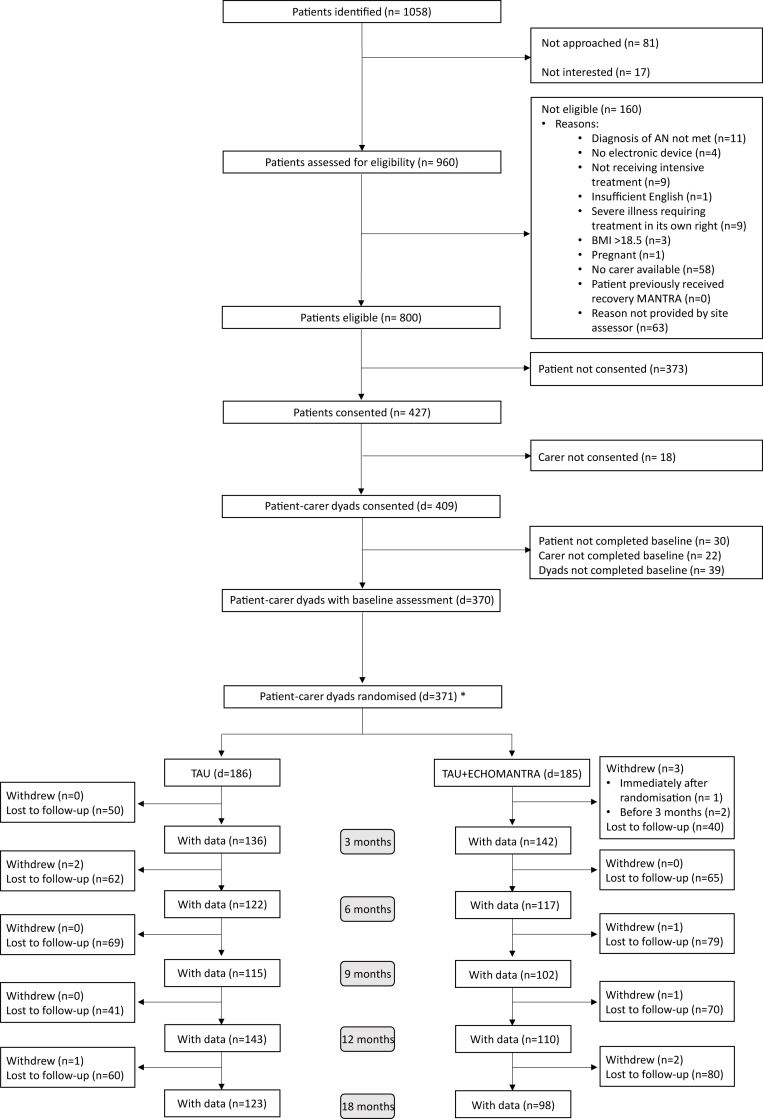
Consort diagram. Diagram describing the flow of participation in the study. ∗Note: The randomised trial sample included one patient who had not completed baseline assessments nor consented to trial participation and thus this patient and their carer was randomized in error. This patient and their carer were therefore withdrawn from the study immediately after randomization, before any treatment commenced. The participant has been noted as an immediate withdrawal within their respective randomised arm and is not included within any analyses due to absent data. Additionally, the trial sample includes three dyads who were randomised despite patients not meeting the BMI inclusion criterion. To maintain randomisation, these are included as part of the intention to treat (ITT) analysis, but subsequently removed from the trial sample as part of a sensitivity analysis.

The study involved a collaboration between 31 treatment centres for patients with anorexia nervosa across the UK (Supplementary Materials 6). The CONSORT diagram (Fig. 1) tracks the longitudinal flow of the patient/carer dyads. Between 1st July 2017 and 20th July 2020, 800 eligible dyads were identified, with 371 dyads randomly assigned to TAU (N = 186 dyads) or TAU + ECHOMANTRA (N = 185 dyads). Because of lower levels of recruitment during the COVID pandemic, a decision was made with the support of the TSC, DMC, and the Funder to stop recruitment in July 2020 at *N* = 371 rather than the target of *N* = 380.

### Study population

The baseline characteristics of the patients and carers recruited are shown in Table 1. As expected, these characteristics were well balanced across trial arms. Patients were predominantly of female sex, of white ethnicity, single, aged 25–26, with no children. Over 50% had post school level academic attainments. The mean illness duration was 8 years. Participants were significantly under-weight on admission to the study with 76% receiving inpatient and 24% day-patient care. Nineteen percent were admitted for involuntary treatment under the Mental Health Act (an additional 17% had had a previous involuntary admission). Self-reported depression and anxiety were common co-morbidities, less common were obsessive compulsive disorder (16%) and autism spectrum disorder (5%). Most carers were white, mothers on average aged 50 years, female (70%), and married. Most were parents (76%) to the patient, although 17% were partners. Most had either a university undergraduate (32.8%) or a postgraduate degree (21%) and were in full time (48.4%) or part time employment (23.1%). Carer characteristics were also comparable across trial arms.

**Table 1:** Patient and carer baseline demographic and clinical variables by trial arm and overall.

Patient variable	TAU = 186 N (%)	TAU + ECHOMANTRA = 184^a^ N (%)	Overall N = 370 N (%)
**Age**			
Mean (SD)	25.1 (8.4)	25.8 (9.4)	25.4 (8.9)
Median (IQR)	22.9 (19.4–27.6)	22.4 (19.6–28.9)	22.5 (19.5–27.8)
**Sex (self-reported)**			
1. Male	11 (5.9)	13 (7.1)	24 (6.5)
2. Female	175 (94.1)	171 (92.9)	346 (93.5)
**Ethnicity**			
1. Asian	4 (2.2)	1 (0.5)	5 (1.4)
2. Black	2 (1.1)	0 (0.0)	2 (0.5)
3. White	176 (94.6)	174 (94.6)	350 (94.6)
4. Mixed	4 (2.2)	8 (4.4)	12 (3.2)
Missing	0 (0.0)	1 (0.5)	1 (0.3)
**Highest completed education**			
1. No Qualifications	5 (2.7)	7 (3.8)	12 (3.2)
2. O Level/GCSE	34 (18.3)	24 (13.0)	58 (15.7)
3. A Level/NVQ	50 (26.9)	67 (36.4)	117 (31.6)
4. Diploma/BTEC	19 (10.2)	23 (12.5)	42 (11.4)
5. University Degree	48 (25.8)	47 (25.5)	95 (25.7)
6. Postgraduate degree	23 (12.4)	16 (8.7)	39 (10.5)
7. Other	6 (3.2)	0 (0.0)	6 (1.6)
Missing	1 (0.5)	0 (0.0)	1 (0.3)
**Marital status**			
1. Married	14 (7.5)	21 (11.4)	35 (9.5)
2. In a relationship and cohabit.	15 (8.1)	15 (8.2)	30 (8.1)
3. In a relationship not cohabit.	14 (7.5)	11 (6.0)	25 (6.8)
4. Single	139 (74.7)	133 (72.3)	272 (73.5)
5. Divorced	0 (0.0)	2 (1.1)	2 (0.5)
6. Separated	2 (1.1)	2 (1.1)	4 (1.1)
7. Widowed	1 (0.5)	0 (0.0)	1 (0.3)
Missing	1 (0.5)	0 (0.0)	1 (0.3)
**Treatment type**			
Inpatient care	143 (76.9)	140 (76.1)	283 (76.5)
Day-care	43 (23.1)	44 (23.9)	87 (23.5)
**Height (cm)—Mean (SD)**	165.4 (7.1) [1]	165.9 (7.7)	165.6 (7.4) [1]
**Clinician reported weight at admission (kg)—Mean (SD)**	40.1 (6.1) [11]	39.6 (6.3) [15]	39.9 (6.2) [26]
**Participant reported weight (kg)—Mean (SD)**	43.4 (6.4) [6]	43.8 (6.9) [5]	43.6 (6.7) [11]
**Lowest weight since eating disorder began (kg)—M (SD)**	36.9 (5.9) [9]	36.6 (6.1) [5]	36.7 (6.0) [14]
**Highest weight ever (kg)—Mean (SD)**	57.6 (12.0) [16]	58.3 (11.4) [18]	57.9 (11.7) [34]
**Years with eating disorder**			
Mean (SD)	7.8 (8.2) [4]	8.4 (8.3)	8.1 (8.3) [4]
Median (IQR)	5.0 (3.0–10.0) [4]	5.5 (3.0–10.0)	5.0 (3.0–10.0) [4]
**Depression diagnosis**			
1. No	70 (37.6)	68 (37.0)	138 (37.3)
2. Yes	114 (61.3)	116 (63.0)	230 (62.2)
Missing	2 (1.1)	0 (0.0)	2 (0.5)
**Anxiety diagnosis**			
1. No	75 (40.3)	75 (40.8)	150 (40.5)
2. Yes	109 (58.6)	109 (59.2)	218 (58.9)
Missing	2 (1.1)	0 (0.0)	2 (0.5)
**OCD diagnosis**			
1. No	157 (84.4)	151 (82.1)	308 (83.2)
2. Yes	27 (14.5)	33 (17.9)	60 (16.2)
Missing	2 (1.1)	0 (0.0)	2 (0.5)
**ADHD diagnosis**			
1. No	182 (97.9)	177 (96.2)	359 (97.0)
2. Yes	2 (1.1)	7 (3.8)	9 (2.4)
Missing	2 (1.1)	0 (0.0)	2 (0.5)
**Autism spectrum disorder diagnosis**			
1. No	173 (93.0)	175 (95.1)	348 (94.1)
2. Yes	11 (5.9)	9 (4.9)	20 (5.4)
Missing	2 (1.1)	0 (0.0)	2 (0.5)
**Panic disorder diagnosis**			
1. No	175 (94.1)	170 (92.4)	345 (93.2)
2. Yes	9 (4.8)	14 (7.6)	23 (6.2)
Missing	2 (1.1)	0 (0.0)	2 (0.5)
**Specific phobia diagnosis**			
1. No	168 (90.3)	175 (95.1)	343 (92.7)
2. Yes	16 (8.6)	9 (4.9)	25 (6.8)
Missing	2 (1.1)	0 (0.0)	2 (0.5)
**Other psychological disorder**			
1. No	152 (81.7)	157 (85.3)	309 (83.5)
2. Yes	31 (16.7)	27 (14.7)	58 (15.7)
Missing	3 (1.6)	0 (0.0)	3 (0.8)
**Treated under mental health act**			
1. Yes—currently	36 (19.4)	35 (19.0)	71 (19.2)
2. Yes—previously	32 (17.2)	30 (16.3)	62 (16.8)
3. No—never	118 (63.4)	118 (64.1)	236 (63.8)
Missing	0 (0.0)	1 (0.5)	1 (0.3)
**Times treated under the mental health act**			
Mean (SD)	1.8 (1.0)	1.8 (1.1)	1.8 (1.0)
Median (IQR)	2.0 (1.0–2.0)	1.0 (1.0–2.0)	2.0 (1.0–2.0)
**Treated under a community treatment order**			
1. Yes—currently	6 (3.2)	6 (3.3)	12 (3.2)
2. Yes—previously	9 (4.8)	6 (3.3)	15 (4.1)
3. No—never	167 (89.8)	170 (92.4)	337 (91.1)
Missing	4 (2.2)	2 (1.1)	6 (1.6)

Carer variable	**TAU N** = **186** N (%)	**TAU + ECHOMANTRA** = **184**^a^**N (%)**	**Overall N** = **370 N (%)**
**Age—Mean (SD)**	50.4 (12.7) [2]	49.9 (12.6) [2]	50.1 (12.6) [4]
**Sex (self-reported)**			
1. Male	55 (29.6)	56 (30.4)	111 (30.0)
2. Female	131 (70.4)	128 (69.6)	259 (70.0)
**The patient is my** …			
1. Spouse	14 (7.5)	17 (9.2)	31 (8.4)
2. Partner	16 (8.6)	16 (8.7)	32 (8.6)
3. Child	140 (75.3)	141 (76.6)	281 (80.0)
4. Sibling	9 (4.8)	4 (2.2)	13 (3.5)
5. Parent	0 (0.0)	3 (1.6)	3 (0.8)
6. Other relative	2 (1.1)	1 (0.5)	3 (0.8)
7. Friend	4 (2.2)	2 (1.1)	6 (1.6)
8. Other non-relative	1 (0.5)	0 (0.0)	1 (0.3)
**Current employment status**			
1. Paid full time employment	90 (48.4)	82 (44.6)	172 (46.5)
2. Paid part time employment	43 (23.1)	47 (25.5)	90 (24.3)
3. Unpaid volunteer work	4 (2.2)	1 (0.5)	5 (1.4)
4. Sick leave	1 (0.5)	1 (0.5)	2 (0.5)
5. Unemployed	1 (0.5)	4 (2.2)	5 (1.4)
6. Student or pupil	2 (1.1)	4 (2.2)	6 (1.6)
7. Retired	20 (10.8)	20 (10.9)	40 (10.8)
8. House wife or house husband	11 (5.9)	15 (8.2)	26 (7.0)
9. Other	13 (7.0)	9 (4.9)	22 (6.0)
Missing	1 (0.5)	1 (0.5)	2 (0.5)
**Highest completed education**			
1. No qualifications	4 (2.2)	3 (1.6)	7 (1.9)
2. O Level/GCSE	32 (17.2)	37 (20.1)	69 (18.7)
3. A Level/NVQ	21 (11.3)	23 (12.5)	44 (11.9)
4. Diploma/BTEC	21 (11.3)	33 (17.9)	54 (14.6)
5. University Degree	61 (32.8)	55 (29.9)	116 (31.4)
6. Postgraduate degree	39 (21.0)	30 (16.3)	69 (18.7)
7. Other	7 (3.8)	2 (1.1)	9 (2.4)
Missing	1 (0.5)	1 (0.5)	2 (0.5)
**English as a first language**			
1. No	3 (1.6)	6 (3.3)	9 (2.4)
2. Yes	182 (97.9)	177 (96.2)	359 (97.0)
Missing	1 (0.5)	1 (0.5)	2 (0.5)
**Ethnicity**			
1. Asian	5 (2.7)	2 (1.1)	7 (1.9)
2. Black	2 (1.1)	0 (0.0)	2 (0.5)
3. White	174 (93.6)	178 (96.7)	352 (95.1)
4. Mixed	2 (1.1)	2 (1.1)	4 (1.1)
5. Other	1 (0.5)	1 (0.5)	2 (0.5)
Missing	2 (1.1)	1 (0.5)	3 (0.8)
**Marital status**			
1. Married	119 (64.0)	120 (65.2)	239 (64.6)
2. In a relationship and cohabiting	27 (14.5)	22 (12.0)	49 (13.2)
3. In a relationship and not cohabiting	9 (4.8)	7 (3.8)	16 (4.3)
4. Single	9 (4.8)	6 (3.3)	15 (4.1)
5. Divorced	17 (9.1)	16 (8.7)	33 (8.9)
6. Separated	2 (1.1)	6 (3.3)	8 (2.2)
7. Widowed	2 (1.1)	6 (3.3)	8 (2.2)
Missing	1 (0.5)	1 (0.5)	2 (0.5)

*Notes.* Square parentheses for continuous summaries represent number of missing entries (out of 370 patients or carers). Where no square parentheses are present for continuous summaries, data are fully observed.

Formal testing of baseline differences was not performed as this is best practice in the analysis of clinical trials (BMJ 1999; 319:185).

a Baseline data was not available for one patient/carer dyad randomised in error.

### Outcomes

**Fig. 2:**
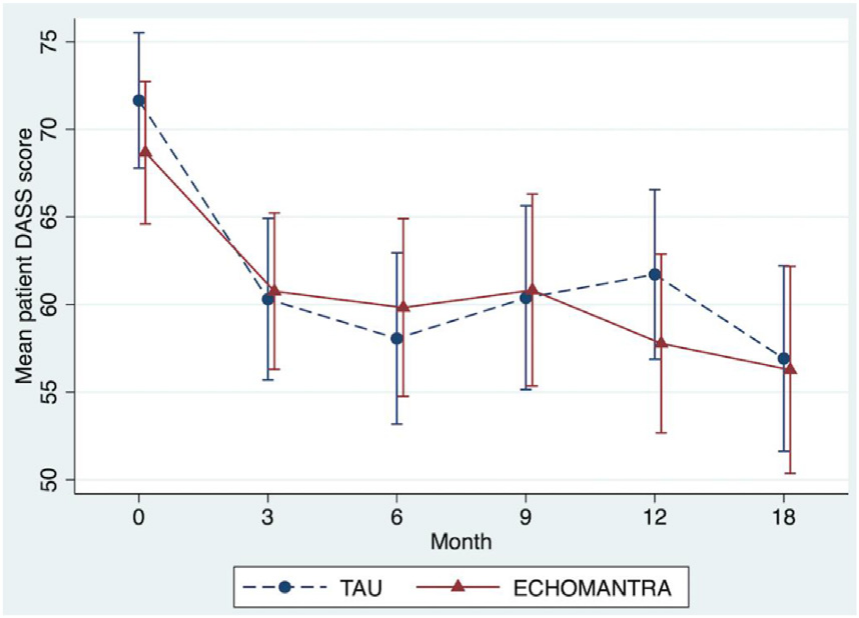
Changes in primary outcome. Profile plots displaying changes in raw mean scores (with 95% confidence intervals) over time by trial arm for patient primary outcome (Depression, Anxiety, and Stress Scales 21: DASS). Scores for the ECHOMANTRA + Treatment As Usual (TAU) group are displayed with the red line. Scores for the TAU only group are displayed with the blue line.

The results of the formal trial arm comparisons for the primary and secondary outcomes are shown in Table 2 (raw data for all outcomes are presented in Supplementary Materials 7). There was no statistical evidence that TAU + ECHOMANTRA changed the primary outcome, patient DASS-21 at 12 months, compared to TAU alone (*p* = 0.87; mean difference 0.48 points; 95% CI −5.36 to 6.33; for an illustration of the raw data see Fig. 2). Multiplicity adjustments were not applied to the *p*-values for the secondary outcomes in Table 2, and the (unadjusted) significant result of WSAS at 18 months is likely to be a random error. Differences between patient groups on secondary outcomes were generally small and nonsignificant (standardised effect size estimates ≤ 0.25). Although there was one exception where the WSAS at 18 months post-randomisation was 3.42 scores worse (higher) after adjusting for missing data biases in the ECHOMANTRA group (*p* < 0.05), this finding does not take account of multiple secondary outcome comparisons and was very sensitive to missing data assumptions (a complete case analysis gave *p* = 0.14). We therefore do not interpret this as evidence for a treatment effect on WSAS.

**Table 2:** Results of the primary and secondary outcome analyses for patients adjusting for missing data biases using multiple imputation.

Patient outcomes	N	Estimated ECHO-MANTRA effect	95% CI	Stan**d.** estimate	Stand. 95% CI	*p*-value
DASS-21—12 m.	370	0.48	(−5.36, 6.33)	0.02	(−0.20, 0.23)	0.87
DASS-21—18 m.	370	1.35	(−5.33, 8.02)	0.05	(−0.19, 0.29)	0.69
EDE-Q—12 m.	370	−0.01	(−0.28, 0.27)	0.00	(−0.21, 0.20)	0.97
EDE-Q—18 m.	370	0.08	(−0.24, 0.40)	0.06	(−0.17, 0.29)	0.62
Patient report BMI—12 m.	370	−0.52	(−1.13, 0.09)	−0.25	(−0.55, 0.04)	0.092
Patient report BMI—18 m.	370	−0.34	(−1.02, 0.34)	−0.17	(−0.50, 0.17)	0.33
WSAS–12 months	370	1.87	(−0.81, 4.55)	0.20	(−0.09, 0.49)	0.17
WSAS–18 months	370	3.42	(0.53, 6.31)	0.37	(0.06, 0.68)	0.021^a^
Motivation to change—12 m.	370	0.17	(−0.51, 0.85)	0.06	(−0.18, 0.31)	0.62
Motivation to change—18 m.	370	−0.09	(-0.82, 0.64)	−0.03	(-0.30, 0.23)	0.81
Ability to change—12 m.	370	−0.10	(−0.72, 0.51)	−0.04	(−0.27, 0.19)	0.74
Ability to change—18 m.	370	−0.20	(−0.87, 0.47)	−0.07	(−0.33, 0.18)	0.56
SDQ—12 m.	370	1.24	(−0.38, 2.86)	0.21	(−0.06, 0.49)	0.13
EQ-5D-3L—12 m.	370	−0.02	(−0.09, 0.05)	0.00	(−0.20, 0.20)	0.62
	**N**	**IRR**	**95% CI**			*p***-value**
CSRI—inpatient hospital days 3 months before the 12 months follow-up	370	0.86	(0.31, 2.41)	–	–	0.77

IRR = incidence rate ratio; m. = months; CI = confidence interval; Stand. = standardised.

a *p* < 0.05.

Similarly, there were no significant differences between trial arms in carer outcomes at 12 or 18 months, which includes symptoms of depression, anxiety, and stress, and skills to manage the illness (Table 3).

**Table 3:** Results of the secondary outcome analysis for carers adjusting for missing data biases using multiple imputation.

Carer Secondary Outcomes	N	Estimated ECHOMANTRA effect	95% CI	Stand. estimate	Stand. 95% CI	*p*-value
DASS-21—12 m.	370	−1.4	(−6.19, 3.38)	−0.06	(−0.25, 0.14)	0.57
DASS-21—18 m.	370	−2.12	(−7.42, 3.18)	−0.09	(−0.31, 0.13)	0.43
CASK–12 months	370	−11.96	(−24.47, 0.55)	−0.29	(−0.60, 0.01)	0.061
CASK–18 months	370	7.08	(−7.02, 21.17)	0.17	(−0.17, 0.52)	0.32

m. = months; CI = confidence interval; Stand = standardised.

### Adherence to the ECHOMANTRA intervention

Only 20% of the dyads met the pre-set criteria for adherence (see Supplementary Materials 8 for detailed information on participation overall). Although the research team had planned for the platform to record frequency of usage of the videos or the written materials, the platform developers experienced technical issues to implement this feature. In accordance with the funder, the TSC and the DMC, the study team decided to progress regardless of the resolution of this problem, to avoid delaying the start of the trial.

### Results of the sensitivity analyses

To assess the effect of treatment receipt, as opposed to treatment assignment, on the primary outcome patient DASS-21, the complier average causal effect (CACE) was estimated. No evidence of a statistically significant effect of treatment receipt was observed (*p* = 0.84), although the effect estimate (3.11 points, 95% CI −26.92 to 33.13) was increased relative to the ITT estimate (0.48 points). Results were little affected by the removal of three patients with atypical anorexia nervosa, who had a BMI >18.5 kg/m^2^ at the point of recruitment (estimated difference 0.28 points, 95% CI = −5.58 to 6.15, *p* = 0.92). Finally, a re-analysis of the primary outcome after excluding all data collected after the onset of the COVID-19 pandemic (11th March 2020) did not change the pattern of findings (excluding *n* = 33 patients randomised post-COVID, estimated difference 1.71, 95% CI = −7.15 to 10.57, *p* = 0.70).

### Cost effectiveness analysis

Table 4 provides a summary of the economic evaluation. While quality of life improved in both groups (see Table 2), QALY gains did not significantly differ across the TAU and ECHOMANTRA groups (*p* = 0.10; mean difference–0.06 QALYs; 95% CI −0.12 to 0.01). The additional costs of providing ECHOMANTRA were £298 per patient (Supplementary Materials 4). The use of services at 12 months was substantially lower in both groups but with no overall significant differences between groups (see Supplementary Materials 9). Overall, one year mean costs per patient from both the health system perspective and wider societal perspective were not significantly different across groups (health perspective *p* = 0.32; mean difference £5948; 95% CI −£6297 to £17,786, societal perspective *p* = 0.61; mean difference £3351; 95% CI −£9253 to £15,371). These results remained robust when performing bootstrap replications of costs and outcomes in order to generate 95% CIs for incremental cost per QALY gained.

**Table 4:** Cost per additional QALY gained (health and societal perspectives).

Outcome	ECHOMANTRA (N = 184) Mean, SD	TAU (N = 185) Mean, SD	Mean Difference (95% CI)^a^	*p*
Total health system perspective cost	85,902 (59,758)	79,954 (55,086)	5948 (−6,297, 17,786)	0.32
Total societal perspective cost	96,839 (62,348)	93,488 (59,355)	3351 (−9,253, 15,371)	0.61
QALYs	0.529 (0.299)	0.530 (0.313)	−0.001 (−0.067, 0.060)	0.96
QALY change	0.008 (0.317)	0.064 (0.327)	−0.059 (−0.122, 0.010)	0.10
ICER (cost per QALY gained) (95% CI) health system perspective	Dominated by TAU (Dominated, 549,899)^b^			
ICER (cost per QALY gained) (95% CI) societal perspective	Dominated by TAU (Dominated, 513,355)^b^			

a Bias corrected accelerated bootstraps.

b Confidence intervals from 1000 bootstrapped paired samples of costs and outcomes.

**Fig. 3:**
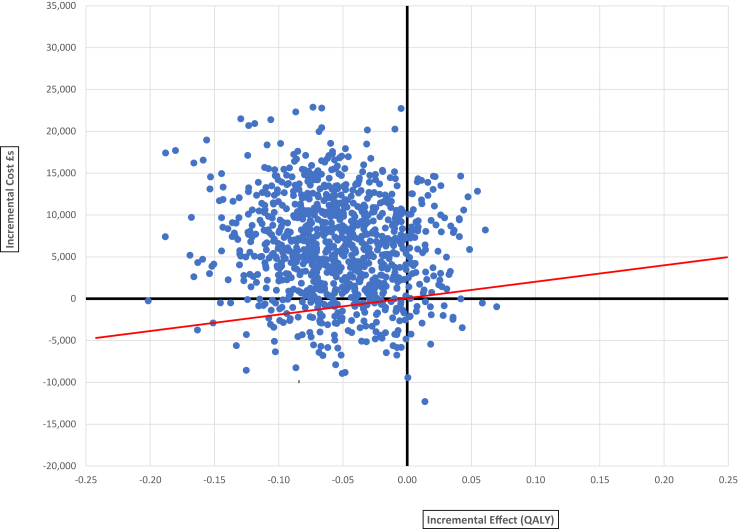
Cost-effectiveness plane comparing ECHOMANTRA + TAU compared to TAU from a health system perspective. The cost effectiveness plane shows simulated outputs from probabilistic sensitivity analysis for ECHOMANTRA + Treatment as Usual (TAU) compared to TAU only. The vertical axis indicates the incremental cost of ECHOMANTRA + TAU from a health system perspective and the horizontal axis the incremental impact on quality adjusted life years (QALYs) in each simulation. The red line represents a willingness to pay threshold of £20,000 per QALY gained, with only simulations below the line being under this threshold.

Cost effectiveness planes from both the health system and societal perspectives (Fig. 3 and Supplementary Materials 10) show that 77% (health system perspective) or 64% (societal perspective) of bootstrapped iterations had both lower QALYs and higher costs compared to TAU. These results did not change when analysis based on completer cases only were used in sensitivity analyses. However, the level of engagement with ECHOMANTRA may have an impact on the economic case. When the differences in costs and quality of life outcomes were explored between the 36 ECHOMANTRA patient/carer dyads that had at least four online group sessions and the remaining 148 patient/carer dyads who attended fewer sessions, it was found that costs were lower and quality of life outcomes greater in the ECHOMANTRA completer group (Supplementary Materials 11).

### Adverse events

Five patients died (3 in TAU and 2 in ECHOMANTRA) during the 18 months of monitoring of either suicide (*n* = 2) or physical health problems caused by anorexia nervosa (*n* = 3). Details on health deterioration/relapse are provided for patients (mainly weight loss) in Supplementary Materials 11 and for carers in Supplementary Materials 12.

## Discussion

The aim of this study was to examine if the ECHOMANTRA intervention could be a useful augmentation to aftercare following intensive treatment for anorexia nervosa. We found no evidence that the intervention produced any group differences in the primary outcome (patient depression, anxiety and stress symptoms at 12 months) or secondary outcomes (eating psychopathology, self-reported BMI and social functioning at 12 and 18 months) in patients or carers (distress and caregiving skills at 12 and 18 months). ECHOMANTRA was also not found to be more cost effective compared to TAU; however, exploratory analyses suggested that levels of engagement may have played a role, such that lower costs and greater quality of life were seen in those who engaged with the ECHOMANTRA intervention.

Adherence to the intervention, defined as actively participating in a minimum of four online groups (for both patients and carers in the dyads), was very low (20%). It was not possible to access data for the usage of the other components of the intervention (i.e., written psychoeducation materials and videoclips), although qualitative studies suggest that these were more widely used.^28^

The current study was a superiority trial. We therefore did not set out to assess the equivalence of the TAU and ECHOMANTRA conditions. The absence of any detected differences between randomisation groups does not necessarily mean that actual treatments were equivalent, in particular when there was only limited uptake of the ECHOMANTRA treatment. The results of this trial do not fully align with the preceding proof of concept studies that were conducted on individual components of the ECHOMANTRA intervention.^8^^,^^10^ There are several explanations that might account for the failure to find a superior impact of ECHOMANTRA on patient and carer outcomes in this study. The treatment setting (intensive care) is notoriously associated with high levels of ambivalence towards change in patients with anorexia nervosa, as well as feelings of coercion and exhaustion.^29^ There is also an increased focus on medical health rehabilitation, which might lead patients to disregard efforts to engage in psychological treatments and increase a sense of connection with others sharing similar problems. This might have motivated a lack of engagement in online groups. Furthermore, the use of digital resources, with no regular and personalised guidance, might have reinforced the feeling of being “pigeonholed”^30^ rather than being seen as individuals with unique needs.^29^ An additional and related factor, which might account for the non-alignment of findings to the previous studies, is that the patient population in the current study were older, with a longer duration of illness, and with a larger proportion having mandated treatment than the population included in the CASIS study.^8^ The patient population in the iMANTRA study were highly selected (only 19% of those approached agreed to participate) and were slightly younger.^10^ Older age and longer duration of illness have been associated with a worse prognosis^31^ and might in part explain poor adherence to ECHOMANTRA.

There were also differences in the interventions. For example, in the CASIS study the intervention (for carers alone) was open to more than one carer (for example 144 mothers and 81 fathers were included). This is relevant as a fragmented family approach is associated with poorer outcomes.^32^ Also, the telephone guidance offered in the CASIS study was individualised and shaped to the needs of patients and their carers. The intervention in the iMANTRA study was also personalised with weekly email guidance.

Finally, and importantly, since the time when the CASIS and iMANTRA studies were published, there have been changes in the form and content of “usual” treatment for anorexia nervosa. An example is the wider access to self-management resources for their carers. This has been highlighted also by participants in the “control arm” of the trial, who reported access to resources related to ECHOMANTRA (e.g., written materials and carer support groups) in the public domain themselves.^33^ This dissemination is possibly explained by the quality standards for inpatient care set by the Royal College of Psychiatrists in the UK. For example, the need to involve carers in planning following hospital discharge and to signpost or deliver supplementary support for carers was added in the 2017 guidelines^34^ and expanded in 2021.^35^

This study has some important limitations. Participants' drop-out rates, especially in the intervention group, were high. Unfortunately, this problem is prevalent in treatment studies of eating disorders. For example, a recent systematic review, specifically focusing on aftercare interventions for patients with eating disorders (N = 7, all RCTs)^6^ highlighted drop-out rates ranging from 37% to 57% for pharmacological interventions, and from 30 to 60% for completion of digital self-help programmes or assessments related to the use of these programmes at 12 months. The review also indicated relapse rates ranging from 53% to 22% for cognitive behaviour therapy and suboptimal completion rates (41.7%) for acceptance and commitment therapy. These findings largely overlap with those found in the TRIANGLE trial and reiterate the importance of considering strategies to strengthen uptake, adherence, and retention in eating disorder treatment trials. With the overall goal to gain an understanding of possible strategies, the research team conducted several qualitative studies of participants' feedback in the TRIANGLE trial.^28^, ^33^ Overall, feedback from the patient group suggested that the materials offered as part of ECHOMANTRA did not fully represent their diversity in terms of illness and social characteristics. This is echoed by patients’ narratives of increased need for personalised care.^29^ The main reason carers gave for non-engagement was a lack of time. This aligns with previous studies which have reported more than 40 h of care provision per week.^36^^,^^37^ Also, by recruiting only one carer, the potential for a shared and integrated approach within the family might have been diminished. A related missed opportunity is that the intervention was not integrated with either the inpatient or outpatient clinical teams.

A further possible limitation is that a large proportion of the ECHOMANTRA group chose to disengage from the study after attending a cycle of online groups (*N* = 8) at 16–20 weeks. Several studies have found that early change is associated with a better outcome,^31^^,^^38^ and therefore the identification of a differential impact of ECHOMANTRA may have been hindered by losing this group to follow up. An additional limitation is that outcomes were only assessed by means of self-reported questionnaires (including BMI, which was self-reported to reduce burden on clinicians). This was mostly due to technical problems in recording usage of the intervention materials automatically through the study's website. Also, quality of life and self-report service utilisation data were only collected at baseline and 12 months and any potential short-term improvements in quality of life or changes in service utilisation would have been missed. The conclusions of the economic analysis might have differed if impacts on carer quality of life and service utilisation had also been considered.

Despite these limitations, the results from TRIANGLE are significant for several reasons. TRIANGLE is the largest clinical trial involving patients with anorexia nervosa, to date. It was conducted with relatively low resources (i.e., two research assistants) and it continued to be implemented during COVID-19 in contrast to other clinical trials that had to be discontinued or paused during that time. The finding that quality of life and cost effectiveness were greater in the ECHOMANTRA completers signals that ECHOMANTRA might have produced some form of clinical benefit if patients engaged with the minimum dose. Moreover, it is likely that the statistical differences between trial arms were diminished due to changes in usual care practices that more commonly involved carers and resources similar to ECHOMANTRA; although this poses difficulty from a research perspective, it also highlights the scalability and real-world implementation opportunities of these resources.

The lack of a superiority effect of the experimental condition over the control condition indicates that greater efforts should be made to personalise aftercare for patients with anorexia nervosa, with the overall goal to improve adherence and sustain motivation to change over time. This could be achieved by implementation of personalised guidance and accountability. Also, it might be possible to collect patient data over time to identify triggers for unhelpful behaviours and deploy therapeutic strategies at those difficult times (i.e., also defined as “just-in-time interventions”).

Overall, the findings of the TRIANGLE trial suggest that there needs to be a greater emphasis on more active, inclusive, integrated, and personalised forms of aftercare to sustain behaviour change gains made within the inpatient setting and to lessen the impact of the illness on carers. Moreover, there is a need to consider the potential added burden of adjunctive tools and resources, which might inadvertently dampen engagement among patients and carers despite their propensity to confer benefit. Offering greater integration of adjunctive support with usual care may reduce perceived barriers and enhance uptake and engagement.

In conclusion, although adding ECHOMANTRA to treatment as usual did not significantly enhance clinical outcomes, this might have been because of poor uptake of some of the novel aspects of the intervention in combination with increased dissemination of some of the more established aspects as part of usual care practices. Strategies to maximise patients’ engagement with the ECHOMANTRA treatment components might be associated with greater effectiveness and cost-effectiveness of the intervention.

## Contributors

JT, VC, SA, PM, US, JA and SL contributed to conceptualization and funding acquisition. JT, VC, KR, DCB and PM contributed to investigation. JT, VC, SL and SA contributed to methodology. VC, KR and DCB were responsible for project administration. JT, VC, and PM contributed to supervision. JT, VC, SA, SL, JL, DM, KR and PM contributed to writing—original draft. JT, VC, SA, SL, JL, DM, KR, PM, DCB, US, JA and AH contributed to writing—review and editing. All authors listed approved the final version and agreed to be accountable for all aspects of the work. SL, DM, EB and JL coded the data and contributed to formal analysis. DCB and PM contributed to formal qualitative analysis. JT, SL, JL, VC, SA, KR have accessed and verified the underlying data.

## Data sharing statement

The trial protocol was published.^12^ The full study report and anonymised participant level dataset will be made available to other researchers three years after the publication of the primary outcomes paper, upon reasonable request made to the corresponding author, to achieve aims of an ethically approved proposal.

## Declaration of interests

JT has written the book, ‘Skill-based Learning for Caring for A Loved One with an Eating Disorder’ which was given to carers in this study. US receives royalties from Routledge for various patient workbooks on eating disorders. SA received support from the HTA for conference registration fees for the London Eating Disorders International Conference (2023). VC receives support from the University of Padova, and has received grants from the Medical Research Council, and the British Academy. JT, US and SL receive salary support from the NIHR Maudsley Biomedical Research Centre (BRC) for Mental Health, South London and Maudsley NHS Foundation Trust and Institute of Psychiatry, Psychology and Neuroscience, and King's College London. SL is also supported by the (NIHR) Applied Research Collaboration (ARC), South London. The views expressed in this publication are those of the authors and not necessarily those of the National Health Service, the NIHR or the UK Department of Health. There are no other conflicts of interest to disclose.
