# Supplementary material for: Efficacy and cost-effectiveness of a digital guided self-management intervention to support transition from intensive care to community care in anorexia nervosa (TRIANGLE): pragmatic multicentre randomised controlled trial and economic evaluation
Source: eClinicalMedicine. 2024 May 27;73:102645. doi: 10.1016/j.eclinm.2024.102645 (PMC11152892; doi:10.1016/j.eclinm.2024.102645)
Supplement: Supplementary Materials [file mmc1.pdf]

Efficacy and cost-effectiveness of a digital guided self-management intervention to support transition from intensive care to community care in anorexia nervosa (TRIANGLE): pragmatic multicentre randomised controlled trial and economic evaluation

Supplementary materials

Supplement #	Description
1	Protocol amendments
2	Additional details about study measures
3	Unit costs used in the economic evaluation (2022 £s)
4	ECHOMANTRA Intervention costs (£s 2022)
5	List of participating sites
6	Summaries of patient outcome scales by assessment timepoint and trial arm
7	Summary of online forum sessions attended by patient and carer participants throughout the intervention period by group type
8	Mean Difference in Service Utilisation Per Participant at 12 Month Follow Up (previous 3 months) (Imputed dataset)
9	Cost-effectiveness plane – societal perspective
10	Comparison of previous 3-month costs at 12-month follow up and QALYs gained ECHOMANTRA group who did or did not complete at least 4 online group sessions (Imputed dataset) (2022 £s)
11	Patient adverse events by trial arm and overall
12	Carer adverse events by trial arm and overall

Supplement 1.

Protocol amendments

Amendment	Changes	From Protocol	To Protocol N, Version	Date Effective
Substantial amendment 1	Change to intervention delivery<br>1:1 guidance sessions replaced with 8 online moderated and facilitated group sessions for patients and carers.<br><br>Change to measures<br>Patient *Difficulties in Emotion Regulation Scale*, Carer *Parents Versus Anorexia Nervosa* and *Accommodation and Enabling Scale for Eating Disorders* questionnaires were removed to reduce participant burden.<br><br>Change to inclusion criteria<br>- Patients should either be admitted for inpatient care or should be attending day care for a minimum of 4 days/week at the time of consenting (previously unspecified how long patients should be attending day care for)<br>- Patients previously only included if they suffered from anorexia nervosa, criteria changed to include subclinical/atypical anorexia nervosa (BMI > 18.5)<br>- Clarification that patients must be able to access an electronic device and the internet to log onto the study platform<br>- Informed consent should be signed within 2 months from admission (changed from 1 month)<br><br>Addition of sites<br>- North Essex Partnership NHS Foundation Trust.<br>- Cardiff and Vale University Health Board (NHS).<br>- 2GETHER NHS Foundation Trust<br>Addition of study documents<br>- Eligibility criteria check list for local investigators and CSO	1	2<br>(Protocol 2, 09/02/17)	27/3/2017

	- Pre- and Post-discharge clinical team survey<br>- Mentor's questionnaires			
Non-substantial amendment 1	Addition of sites<br>Berkshire Healthcare NHS Foundation Trust<br>South West London and St George's Mental Health NHS Trust	NA	NA	03/05/2017
Non-substantial amendment 2	Addition of sites<br>- Surrey and Borders Partnership NHS Foundation Trust	NA	NA	31/05/2017
Substantial Amendment 2	Change to study documents<br>- Address of participant added to consent forms for reimbursement by cheque.<br>- Question added to the consent form asking whether participants are happy to be contacted about other research projects in the future.<br>- Patient information sheet (PIS) typo corrected: header "Version 3_0902" renamed to Version 4, 09/02/2017"<br><br>Change to measures<br>Monthly surveys to clinicians now include information about Serious Adverse Events (SAE's)<br><br>Change to consenting process<br>- To improve data security, changed process of sending consent forms from non-NHS sites to the research team at King's College: consent forms now password protected and attached in a normal email to the research team.<br><br>Change to participant reimbursement<br>Patients now reimbursed by cheque every 6 months during the study rather than at the end<br><br>Addition of new sites<br>- Berkshire Healthcare NHS Foundation Trust<br>- South West London and St George's Mental Health NHS Trust	2	3 (Protocol 3, 07/07/17)	20/7/2017

	- Surrey and Borders Partnership NHS Foundation Trust<br><br>Change to measures<br>Social Identity Map exercise added to the TRIANGLE website.			
Non-substantial amendment 3	Change to consenting process<br>Clarification that carers can consent from a distance (by email/post). Postage will be reimbursed by the research team.	NA	NA	16/08/2017
Non-substantial amendment 4	Addition of new site<br>- Devon Partnership NHS Trust	NA	NA	11/10/2017
Substantial Amendment 3	Change to data collection<br>Due to participant difficulty in completing the *Client Service Receipt Inventory* and *Social Identity Map*, research assistants will now provide telephone guidance to complete these assessments.<br><br>Change to measures<br>- Questions regarding perceived treatment credibility and acceptability added to monthly questionnaire for ongoing evaluation of the intervention.<br>- Sites will now send medical records including blood test results, blood pressure, pulse, temperature, and saturation via encrypted email to the research team at baseline and when the patient is discharged.<br><br>Change to consent forms<br>Patient and carer consent forms revised to include the above changes.	3	4<br>(Protocol 4, 28/09/17)	20/11/2017
Substantial Amendment 4	Change to study documents<br>A typo in the footer of the Patient Information sheet was rectified. It was a discrepancy between the front-page date and the footer date.<br><br>Change to measures<br>Addition of other eating disorder related measures for patients willing to provide additional data	4	5<br>(Protocol 5, 08/11/17)	19/1/18

Substantial Amendment 5	Change to protocol<br>- Serious Adverse Events defined to specify whether they would be considered "related", "unrelated", "expected" or "unexpected" to clarify which kind of events will be recorded only and which events will need reporting to the REC.<br>- Procedure for dealing with protocol violations added to protocol: protocol violations are recorded by the research assistants with justifications. These are discussed during regular meetings with the statistician and based on those discussions the study team consider whether an update to the protocol will be necessary.<br>- Following under-recruitment from recruiting sites, TRIANGLE research assistants will now visit the participating sites on a regular basis to assist with recruiting and consenting patients.<br>- BMI inclusion criteria now applies at the time the patient is approached (not at consent).<br>- Exclusion criteria specifying that patients not admitted to inpatient/day-care for a minimum of 4 days/week applies at the time they begin the study (not at consent).<br><br>Staff/research team changes<br>- Changes to the Data Monitoring Committee (DMEC) membership Dr John Morgan has replaced Prof. Hubert Lacey as the DMEC Chair. Dr Eric Johnson Sabine has replaced Dr Robert Palmer as Psychiatrist.<br>- Principal Investigator change at Barnet, Enfield and Haringey Mental Health Trust - from Dr Lorna Richards to Minna Raikkonen.<br><br>Change to measures<br>Additional questions added to feedback forms to be completed by patients, carers and mentors receiving ECHOMANTRA<br><br>Change to study documents	5	6 (Protocol 6, 01/02/18)	29/5/18

	- Patient information sheet (PIS) typo corrected: header "Version 3_0902" renamed to Version 4, 09/02/2017"<br>- Minor changes to consent forms following feedback from clinical teams that participants often make mistakes and that making the form clearer could help to reduce delays with consenting.<br>- Additional study flyer to help with recruitment.<br><br>Addition of new sites<br>- Berkshire Healthcare NHS Foundation Trust<br>- South West London and St George's Mental Health NHS Trust<br>- Surrey and Borders Partnership NHS Foundation Trust			
Non-substantial amendment 5	Clarification of participant reimbursement<br>Clarification that the total reimbursement is £60 for patients and £60 for carers<br><br>Changes to study documents<br>- Typo Eligibility Checklist<br><br>Staff/research team changes<br>Principal Investigator change at South West London and St George's Mental Health NHS Trust – from John Adlam to Jennifer Walker.	6	7<br>(Protocol 7, 25/07/18)	31/07/2018
Non-substantial amendment 6	Study flyer Typo<br>A typo in the study flyer was rectified. It was a discrepancy between the front-page date and the footer date.<br>- Corrected: header "Version 2_21.05.2018" renamed to Version 3, 19/09/2018"	NA	NA	19/09/2018
Substantial amendment 6	Principal investigator change<br>Principal Investigator change at Northumberland, Tyne and Wear NHS Foundation Trust - from Dr Mark Willis to Dr Caroline Reynolds.<br><br>Addition of new participating sites<br>The following new participating sites have agreed to take part in the study:	7	8<br>(Protocol 8, 19/09/18)	27/12/2018

-New Market House Health Care Ltd (non-NHS site)
-Cygnet Hospital Ealing, Cygnet Health Care (non-NHS site)
-Cardinal Clinic (non-NHS site)

Study checklist changes
-Changes in inclusion criteria: Informed consent will be signed at any time whilst the patient is admitted into hospital and up to 4 weeks after discharge.
- Following feedback from clinicians, age also extended to 16 years old so the team can recruit from adolescent units
-Change in exclusion criteria: The patient will need to be admitted into hospital or attending day care for a minimum of 3 days/week when they are consented in the study.
-Change in recruitment into trial: Study team will be involved in all the recruitment stages.

Addition of electronic consent for carers
- Due to consenting process of carers delaying randomization, carers can now provide consent electronically via the study website.

Changes in measures obtained from the clinical team and from patients
-The clinical teams will no longer be asked to provide monthly updates from patients. Basic clinical information (e.g., BMI, admission/discharge date) will be collected from the clinical teams only at baseline.
-A brief monthly update questionnaire will be completed by patients instead.

Carer/family involvement questions
Addition of 9 questions related to involvement of family members and carers to be asked to the participating sites.
This brief questionnaire will be completed at twice (i.e., at present and 18 months).

Research perception questions
Addition of 6 questions related to research perceptions. These questions will be asked to all our participating sites
once.

	Study flyer changes<br>Changes in study flyer for patients to make it clearer.<br><br>Study flyer for carers<br>Addition of study flyer for carers.<br><br>Patients-carers joint sessions change<br>The content of the joint sessions will be delivered via the study website through an online group format instead of using skype.<br><br>Advertisement of the study on social media<br>The study will be advertised on social media (e.g., Facebook, twitter).<br><br>Recruitment of participants in the community<br>Participants will also be recruited in the community if they meet the inclusion criteria (i.e., admitted to hospital or attending day care for a minimum of 3 days/week at the start of the study).			
Non-substantial amendment 7	Addition of new site<br>New site has agreed to take part in the study as a participating site. The new site is:<br>Cambridgeshire and Peterborough NHS Foundation Trust	Version 8 Dated: 19/09/2018	Version 9 Dated: 17/01/2019	18/02/2019
Non-substantial amendment 8	Collection of patient BMI from clinical teams<br>-A member of the clinical team at the hospital will be asked to provide patient's BMI measurement at baseline and every 3 months up to patient's discharge.<br>Please note that: a) BMI is already collected as part of routine care b) This data will be collected for research purposes c) There is ethics approval to request this data from the medical notes.<br>d) Clinical teams were asked to provide this data monthly from the beginning of the study. As part of Amendment No. 6 and to reduce burden on clinical teams, this was changed to be provided at baseline only. However, the team has found that it is difficult to collect this data from participants (e.g.,	Version 9 Dated: 17/01/2019	Version 10 Dated: 26/04/2019 Atypical	28/06/2019

	sometimes not aware of BMI as part of treatment for anorexia nervosa) and thus, would like to ask again the clinical teams to provide this data at baseline and every 3 months up to patient's discharge.<br><br>Recruitment extension date:<br>Study Funder (i.e., NHRI (National Health Research Institute) has approved extension of recruitment end date until 31st March 2020 instead of the initially proposed date 31st March 2019<br><br>Principal investigator change:<br>Principal Investigator change at Tees, Esk and Wear Valley's NHS Trust – from Katie Bell to Helen McLay.<br><br>Typo age inclusion criteria<br>We would like to amend the age specified in the inclusion criteria. The approved inclusion age is 16. This was approved in amendment No.6.<br><br>There is a mistake in the study protocol. The inclusion criteria age should read "Patients aged 16 years or over" instead of "patients aged 17 years or over".<br><br>Addition of gift vouchers to the reimbursement of participants<br>We would like to offer to participants the option of gift voucher reimbursement as an alternative to the current cheque reimbursement.			
Non-substantial amendment 9	Principal Investigator changes:<br>Tees, Esk and Wear Valley's NHS Trust – from Helen McLay to Nicholas Wolstenholme.<br>NHS Grampian from Jane Morris to Louise Johnston.	-	-	23/10/2019
Non-substantial amendment 10	Extension to recruitment end date<br>From 31st March 2020 to 31st May 2020.<br><br>Change to statisticians listed in the protocol<br>From statistician Emily Robinson to Rachel Holland and Health Economist from Jennifer Beecham to Eva Bonin	Version 10, dated 26/04/19	Version 11, dated 30/10/19	18/11/2019

Substantial amendment 7	Additional qualitative data collection<br>- To obtain participant feedback on their participation in the TRIANGLE trial and to gather further information regarding patients' views on transition from intensive treatment, we will:<br>- Invite a selected subgroup of patients and carers (up to n=22 patients and n=22 carers, selected based on pre-specified criteria) who were randomised to the ECHOMANTRA + TAU intervention group to participate in an interview about their experience of the TRIANGLE trial and intervention. The maximum number of participants is based on the high likelihood of reaching data saturation on the topic investigated, among the study population, with less than 22 participants.<br>- Invite a selected subgroup of patients and carers (up to n=22 patients and n=22 carers, selected based on pre-specified criteria) who were randomised to the TAU only group to participate in an interview about their experience of transitioning from the intensive treatment (inpatient or day care) they were receiving when initially recruited to the trial back to the community. The maximum number of participants is based on the high likelihood of reaching data saturation on the topic investigated, among the study population, with less than 22 participants.<br>- Participants will be recruited who are between the 6 months and 12 months timepoints in their study participation so that they have had enough time to offer a well-formed opinion of what it meant to them to have participated in the study and to have transitioned from intensive treatment. To explore a range of opinions participants will be recruited purposively across study sites according to recruitment site location, gender, age, patient's eating disorder severity (>7 years of illness or below 3 years of illness) and carer's relationship to the patient. Sites will not be involved in the selection of participants. A King's College London researcher, one of the project's Co-	Version 11, dated 30/10/2019	Version 12, 28/01/2020	09/03/2020

	Investigators will contact the participants to see if they are willing to take part in the additional interview. Interviews via Skype voice call or by telephone. The interview will last for up to 1 hour and will consist in a pre-specified list of questions. Data will be typed up, allocated an ID number for confidentiality purposes and then analysed.<br>- An additional 3 process questions added to the participant feedback form, to be asked to participants in the intervention group at 6 and 12 months in the study.			
Substantial amendment 8	Changes to recruitment procedure due to Covid-19 pandemic<br>During the COVID-19 period, TRIANGLE sites remaining open to recruitment will become Patient Identification Centres (PICs). Local research staff will no longer be required to screen patients. Any staff who identify patients interested in triangle, or if patients hear about the study through flyers on the wards, patients can directly contact the TRIANGLE research assistants who will screen and consent patients into the trial remotely using telephone/encrypted email.	Version 12, dated 28/01/2020	Version 13, dated 27/03/2020	14/04/2020
Substantial amendment 9	Re-consenting of patients for the collection of their Hospital Episode Statistics (HES) data from NHS digital.<br>Information sheets and consent forms updated relating to HES data collection, due to previous oversight. Our Data Monitoring Committee also advised collecting data on impact of Covid-19 on our participant cohort.	Version 13, dated 27/03/2020	Version 14, dated 20/05/2020	02/07/2020
Non-substantial amendment 11	Correction of two errors in the protocol regarding the assessment schedule and study extension (31/10/2021 - 31/08/2022)<br><br>Correction of typographical errors in study documents<br>The protocol states that the Motivational Ruler measure was collected at 3, 6, 9, 12 and 18 months, however, it was not intended to be collected at 9 months and there is no variable in the study database for this measure at 9 months. This was an error in the protocol and has now been corrected. Carer Demographics are on the protocol as only collected at baseline; however, it was possible for carers to change during the study (i.e., a carer could withdraw, and a new carer could consent as a replacement). Therefore, carers	-	-	02/11/2021

	could provide updated demographics at each monthly time point throughout the trial. However, no carers changed in the trial in the end, so this error does not affect any participants.<br><br>Extension to study duration that will not have any additional resource implications for participating organisations<br>We received approval from the NIHR-HTA for a non-cost extension to the timeline of the grant. The original end date of the grant was 31/10/2021 and the new end date for the grant is31/08/2022.	
Non-substantial amendment 12	Extension to study duration that will not have any additional resource implications for participating organisations from 31/08/2022 until 30/11/2022.	18/10/2022

Supplement 2.

Additional details about study measures

Patient measures

Demographics: Demographic features were assessed via a self-report questionnaire which included 17 items related to demographic variables (e.g., sex, ethnicity, highest level of education).

Psychological medical history: Details about patients' psychological medical history were assessed via a self-report questionnaire which included items related to clinical variables (e.g., length of eating disorder diagnosis, comorbidities, treatment under the mental health act).

Eating disorder psychopathology: Eating disorder psychopathology was assessed using the Eating Disorder Examination Questionnaire (EDE-Q; Fairburn & Beglin, 1994). The EDE-Q is a 36 item self-report measure of eating disorder symptoms (dietary restraint, eating concerns, weight concerns, shape concerns) in the past 28 days, on a scale ranging from no days (0) to every day (6). In the main analysis, the global score was used as a marker of eating disorder symptom severity. A higher score indicates greater frequency of symptoms.

Body Mass Index (BMI): Weight and height were primarily assessed via self-report. Clinicians at participating centres were also asked to provide weight and height at admission from clinical records.

Depression, Anxiety and Stress symptoms: Levels of depression, anxiety and stress were assessed using the Depression, Anxiety and Stress Scale (DASS-21; Lovibond & Lovibond, 1995). The DASS-21 is a 21 item self-report measure of depression, anxiety and stress symptoms in the past week, on a scale ranging from 0 (did not apply to me at all) to 4 (applied to me very much, or most of the time). A higher score indicates greater frequency of symptoms.

Social Functioning: Social functioning was assessed via the Work and Social Adjustment Scale (WSAS; Mundt, Marks, Shear, & Greist, 2002). The WSAS is a five-item measure of functional impairment. In this trial the WSAS was used to assess the impact of the eating disorder on the ability to function in the

areas of work, home management, social leisure activities, private leisure activities, and personal relationships. A higher score indicates greater impairment.

Social functioning: Social functioning was assessed via the informant-version of the_Strengths and Difficulties Questionnaire (SDQ; Goodman, 1997). The SDQ is a 25-item informant-rated measure of psychological attributes related to the areas of emotional symptoms, conduct problems, hyperactivity/inattentiveness, peer relationship problems, and prosocial behaviour (in the last six months). In this trial the questionnaire was administered to carers as a measure of social functioning in patients. A total score was calculated using four subscales (excluding prosocial behaviour). For the total score, a higher score indicates greater problems.

Autism Spectrum symptoms: Autistic symptoms were assessed via the Autism Spectrum Quotient (AQ-10; Allison, Auyeung, & Baron-Cohen, 2012): The AQ-10 is a 10-item self-report measure of autistic symptoms, rated on a scale from definitely agree, to definitely disagree. The total score is based on all items. A score >6 indicates high levels of autistic symptoms.

Obsessive Compulsive symptoms: Obsessive compulsive symptoms were assessed via the Obsessive Compulsive Inventory - Revised (OCI-R; Foa et al., 2002). The OCI-R is an 18-item self-report measure of obsessive-compulsive symptoms in the past month. The measure covers six dimensions including checking, washing, ordering, hoarding, obsessing, and neutralizing. Items are rated on a scale from 0 (not at all) to 4 (extremely). A score >21 indicates high levels of obsessive-compulsive symptoms.

Importance to change: Importance to change was assessed via a single-item self-report visual analogue scale, “How important is it for you to change? What score would you give yourself out of 10?” was rated on a scale from 1 (not at all important) to 10 (extremely important).

Ability to change: Ability to change was assessed via a single-item self-report visual analogue scale, “How confident are you in your ability to change? What score would you give yourself out of 10?” is rated on a scale from 1 (not at all confident) to 10 (extremely confident).

Health-related quality of life: Health-related quality of life was assessed using the EQ-5D-3L (Kind, Dolan, Gudex, & Williams, 1998). The ED-5D-3L is a five-item, self-report measure of quality of life across five dimensions including mobility, self-care, usual activities, pain/discomfort, anxiety/depression, which are rated as 1 (no problem), 2 (moderate problem) or 3 (severe problem). Thus, a higher score indicates poorer quality of life in each domain. The scale also includes a visual analogue scale (VAS) which indicates the general health status from 0 (worst health imaginable) to 100 (best health imaginable). In the protocol paper we had indicated that the 5 level version of the EQ-5D would be used. Subsequently however, NICE recommended that the 3-level version continue to be used due to problems with the estimated UK utility weightings for the 5 level version (National Institute for Health and Care Excellence, 2019).

Number of days spent in hospital at 12- and 18-months post-randomisation. Hospital Episode Statistics data were requested from NHS Digital for England-based participants only. However, these data for the full trial period were not available.

Economic measures

Resource utilisation. Health service and other resource utilisation was collected using an adapted version of the Client Service Receipt Inventory (J Beecham & Knapp, 1992). The CSRI is a self-report measure which includes items related to the use of health and other services, as well as productivity losses from lost employment. Participants were asked to recall their use of services and other impacts over the previous three months.

Health-related quality of life: Health-related quality of life was assessed using the EQ-5D-3L (Kind, Dolan, Gudex, & Williams, 1998).

Carer measures

Demographics: Demographic features were assessed via a self-report questionnaire which contained 15 items related to demographic variables (e.g., nature of relationship to the patient, employment status, marital status).

Caregiving skills: Caregiving skills were assessed via the Caregiving Skills Scale (CASK; Hibbs et al., 2015). The CASK is a 27-item measure of caregiving attitudes and behaviours for dealing with eating disorder symptoms.

Carer Depression, Anxiety and Stress symptoms: Carer depression, anxiety and stress symptoms were assessed via the DASS-21 (Lovibond & Lovibond, 1995), as described in the section above on patient baseline measures.

Adherence to the ECHOMANTRA intervention: Participants' adherence to the study intervention was defined as both the patient and carer attending at least four online groups. Participation in the online groups was recorded through the transcripts produced at the end of the group and saved on the study platform. To be counted as participating in an online group, participants had to have posted a message to the group at least once. Thus, participants who joined the group or read the transcript of a group were not captured.

Supplement 3.

Unit costs used in the economic evaluation (2022 £s)

Type of Cost	Unit cost	Unit	Source
GP	£42	Per consultation	Unit Costs of Health and Social Care 2022
Practice Nurse	£8.67	Per consultation	Unit Costs of Health and Social Care 2022 (assume a 10-minute consultation)
Dentist	£53.34	Per consultation	for Provider-Performer and assumes average consultation time 20 minutes
Dietician	£100	Per consultation	Unit Costs of Health and Social Care 2022
Eating Disorder Unit Inpatient	£645	Per night	NHS England National Schedule of NHS Costs 2020-2021
Eating Disorder Unit Daypatient	£261	Per day	NHS England National Schedule of NHS Costs 2020-2021 for outpatient attendances
Inpatient Psychiatric Stays	£469	Per day	NHS England National Schedule of NHS Costs 2020-2021 for other specialist mental health services inpatient stay
Hospital General Inpatient Stays (Long-Stay)	£4,974	Finished Consultant Episode	NHS England National Schedule of NHS Costs 2020-2021 Non Elective (Stays of more than 5 days)
Hospital General Inpatient Stays (Short-Stay)	£985	Finished Consultant Episode	NHS England National Schedule of NHS Costs 2020-2021 Non Elective (Assumes no more than 5 day stay)
Hospital General Outpatient Contact	£235	Per consultation	Unit Costs of Health and Social Care 2022 (Average of NHS National Costs for all Outpatient Contacts)
Hospital A&E attendance	£304	Per visit	NHS England National Schedule of NHS Costs 2020-2021 (average cost for all A&E activity)
Actors, entertainers and presenters	£15.54	Per hour	ONS April 2022 Employee Earnings in the UK 2022
Bakers and Flour confectioners	£13.76	Per hour	ONS April 2022 Employee Earnings in the UK 2022
Nursing Auxiliaries and Assistants	£14.74	Per hour	ONS April 2022 Employee Earnings in the UK 2022
Cleaners and Domestics	£12.36	Per hour	ONS April 2022 Employee Earnings in the UK 2022
Youth and Community Workers	£17.91	Per hour	ONS April 2022 Employee Earnings in the UK 2022
Teaching Assistants	£11.03	Per hour	ONS April 2022 Employee Earnings in the UK 2022
Early Education and Childcare Assistants	£12.20	Per hour	ONS April 2022 Employee Earnings in the UK 2022
Primary Education Teaching Professionals	£24.50	Per hour	ONS April 2022 Employee Earnings in the UK 2022
Medical Radiographers	£27.47	Per hour	ONS April 2022 Employee Earnings in the UK 2022

Receptionists	£12.86	Per hour	ONS April 2022 Employee Earnings in the UK 2022
Community Nurses	£22.85	Per hour	ONS April 2022 Employee Earnings in the UK 2022
Marketing managers	£29.97	Per hour	ONS April 2022 Employee Earnings in the UK 2022
Managers and directors in retail and wholesale	£20.00	Per hour	ONS April 2022 Employee Earnings in the UK 2022
Solicitors and lawyers	£31.14	Per hour	ONS April 2022 Employee Earnings in the UK 2022
Sports coaches, instructors and officials	£16.62	Per hour	ONS April 2022 Employee Earnings in the UK 2022
Researchers, unspecified discipline	£24.44	Per hour	ONS April 2022 Employee Earnings in the UK 2022
Higher Education Teaching Professionals	£30.32	Per hour	ONS April 2022 Employee Earnings in the UK 2022
Generalist medical practitioners	£27.89	Per hour	ONS April 2022 Employee Earnings in the UK 2022
Specialist medical professionals	£43.98	Per hour	ONS April 2022 Employee Earnings in the UK 2022
Physiotherapists	£25.24	Per hour	ONS April 2022 Employee Earnings in the UK 2022
Management consultants and business analysts	£28.13	Per hour	ONS April 2022 Employee Earnings in the UK 2022
Health services and public health managers	£32.56	Per hour	ONS April 2022 Employee Earnings in the UK 2022
Hairdressers and barbers	£11.56	Per hour	ONS April 2022 Employee Earnings in the UK 2022
Beauticians and related occupations	£11.95	Per hour	ONS April 2022 Employee Earnings in the UK 2022
Chartered and certified accountants	£27.99	Per hour	ONS April 2022 Employee Earnings in the UK 2022
Legal secretaries	£14.52	Per hour	ONS April 2022 Employee Earnings in the UK 2022
IT quality and testing professionals	£27.56	Per hour	ONS April 2022 Employee Earnings in the UK 2022
Cooks	£12.46	Per hour	ONS April 2022 Employee Earnings in the UK 2022
Chartered architectural technologists, planning officers and consultants	£21.76	Per hour	ONS April 2022 Employee Earnings in the UK 2022
Authors, writers and translators	£19.28	Per hour	ONS April 2022 Employee Earnings in the UK 2022
Project Support Officers	£19.21	Per hour	ONS April 2022 Employee Earnings in the UK 2022
Midwifery nurses	£26.00	Per hour	ONS April 2022 Employee Earnings in the UK 2022
Public relations and communications directors	£42.63	Per hour	ONS April 2022 Employee Earnings in the UK 2022
Wholesaling, retailing, hotel and restaurant staff	£11.63	Per hour	ONS April 2022 Employee Earnings in the UK 2022
Unspecified public sector workers (excluding financial services)	£16.74	Per hour	ONS April 2022 Employee Earnings in the UK 2022

Elementary administrative occupations	£12.81	Per hour	ONS April 2022 Employee Earnings in the UK 2022
Business and financial project management professionals	£32.24	Per hour	ONS April 2022 Employee Earnings in the UK 2022
Marketing associate professionals	£18.69	Per hour	ONS April 2022 Employee Earnings in the UK 2022
CAD, drawing and architectural technicians	£20.03	Per hour	ONS April 2022 Employee Earnings in the UK 2022
Managers and proprietors in agriculture and horticulture	£22.03	Per hour	ONS April 2022 Employee Earnings in the UK 2022
Human resources administrative occupations	£14.64	Per hour	ONS April 2022 Employee Earnings in the UK 2022
Managers and proprietors in unspecified services	£20.55	Per hour	ONS April 2022 Employee Earnings in the UK 2022
Psychotherapists and CBT therapists	£22.86	Per hour	ONS April 2022 Employee Earnings in the UK 2022
Minimum wage rate aged 16-17 (April 2022)	£4.81	Per hour	GOV.UK Minimum Wage Rates for 2022
Minimum wage rate aged 18-20 (April 2022)	£6.83	Per hour	GOV.UK Minimum Wage Rates for 2022
Minimum wage rate aged 21-22 (April 2022)	£9.18	Per hour	GOV.UK Minimum Wage Rates for 2022
Minimum wage rate aged 23+ (April 2022)	£9.50	Per hour	GOV.UK Minimum Wage Rates for 2022

Supplement 4.

ECHOMANTRA intervention costs (£s 2022)

	**Cost £s**
**Group session delivery costs**	
Facilitation of 271 group patient only, carer only or patient/carer group sessions (Band 5)	11,382
Moderation of group patient only, carer only or patient/carer group sessions by volunteers	1,287
Moderation of group patient only, carer only or patient/carer group sessions by paid staff (Band 5)	5,691
Preparation and facilitation of 27 Skype patient, carer and mentor sessions	1,701
Supervision/feedback for all group sessions (mean time per session 30 minutes) (Band 7)	9,553
**Total group session costs**	**29,614**
**Training costs**	
Band 7 delivered training for 5 Training Days 5.5. hours training time for 16 individuals (5 Band 5 and 11 unpaid volunteers)	1,939
Band 5 Trainee training session time costs	1,155
Band 5 Trainee training session time costs	575
**Total training costs**	**3,669**
**Other costs**	
Workbooks	4,600
VIMEO video hosting, sharing and services platform	757
Web-design and platform maintenance/hosting	16, 234
**Total other costs**	**21,591**
**Total costs**	**54,873**
**Cost per trial participant in ECHOMANTRA + TAU group**	**298**

Supplement 5.

List of participating sites (see Supplementary Figure 1. below for map of geographical locations)

01. South London and Maudsley NHS Foundation Trust
02. Cheshire and Wirral Partnership NHS Foundation Trust
03. South Staffordshire and Shropshire Healthcare NHS Foundation Trust
(Became: Midlands Partnership NHS Foundation Trust)
04. Avon and Wiltshire Mental Health Partnership NHS Trust
05. Dorset Healthcare University NHS Foundation Trust
06. Central and North West London NHS Foundation Trust
07. Barnet, Enfield and Haringey Mental Health NHS Trust
08. Leicestershire Partnership NHS Trust
09. Northumberland, Tyne and Wear NHS foundation trust
(Became: Cumbria, Northumberland, Tyne and Wear NHS Foundation Trust)
10. Royal Cornhill Hospital
11. South East Scotland Regional Eating Disorders Unit
12. South West London and St George's Mental Health NHS Trust
13. North Essex NHS Foundation Trust
(Became: Essex Partnership University Trust)
14. 2gether NHS Foundation Trust
(Became: Gloucestershire Health and Care NHS Foundation Trust)
15. Berkshire Healthcare NHS Foundation Trust
16. Oxford Health NHS Foundation Trust
17. Ellern Mede Ridgeway
18. The Priory Hospital Roehampton
19. The Priory Hospital Southampton
20. The Priory Hospital Altrincham
21. The Priory Hospital Bristol
(Withdrew before study start)
22. The Priory Hospital Cheadle Royal
23. Surrey and Borders Partnership NHS Foundation Trust
24. Devon Partnership NHS Trust
25. Ellern Mede Barnet
26. The Priory Hospital Hayes Grove
27. Tees, Esk and Wear Valleys NHS Foundation Trust (Mental Health)
29. Cambridgeshire & Peterborough NHS Foundation Trust
28. Community
30. Newmarket House
31. Cardinal Clinic
32. Orri Eating Disorder Clinic

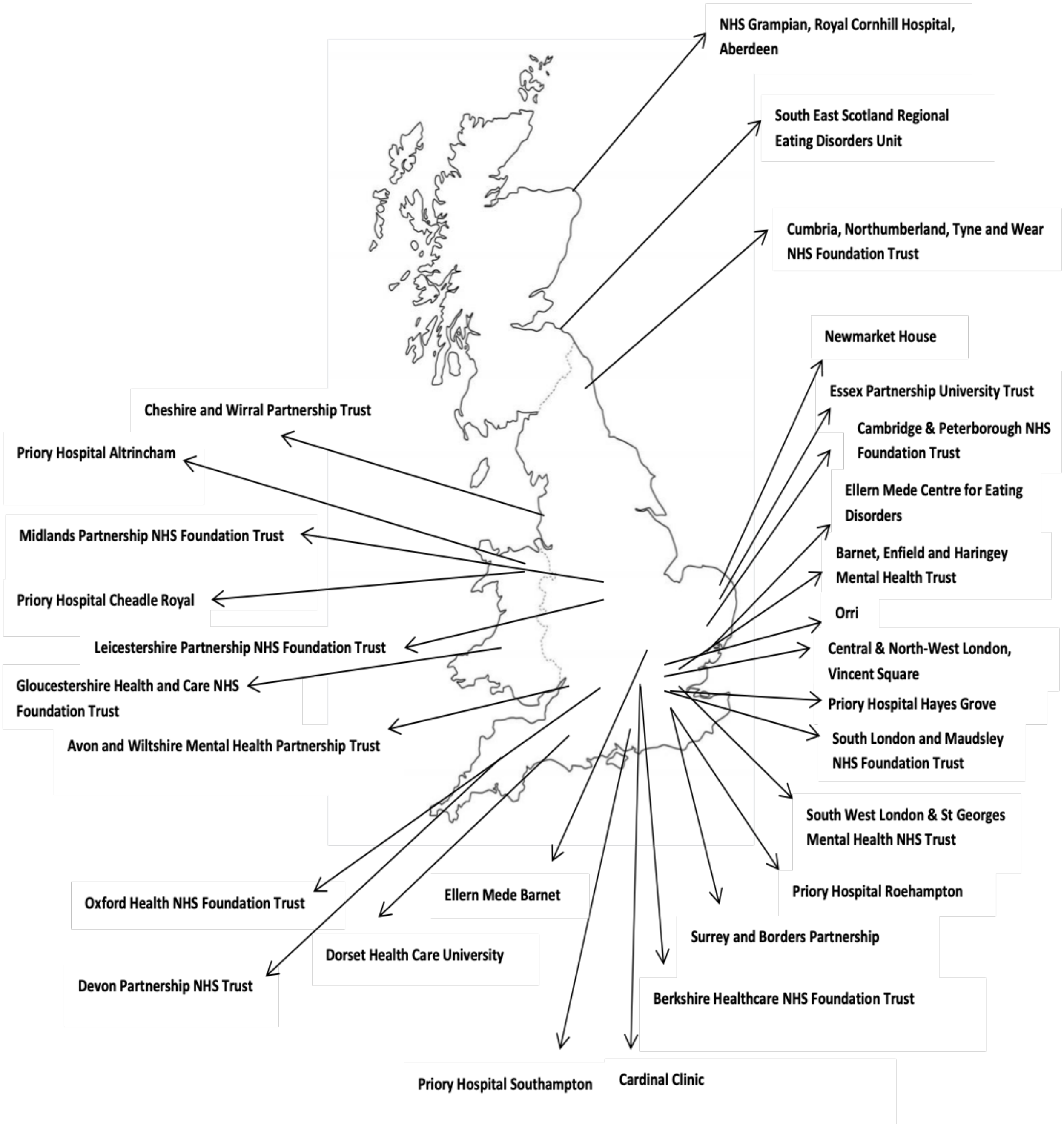

Supplementary Figure 1. *TRIANGLE participating sites by geographical location*

Supplement 6.

Summaries of patient outcome scales by assessment timepoint and trial arm

Outcome measure		Baseline			3 months			6 months			9 months			12 months			18 months		
		TAU	ECHO	Total	TAU	ECHO	Total	TAU	ECHO	Total	TAU	ECHO	Total	TAU	ECHO	Total	TAU	ECHO	Total
**Patient DASS-21**	**N**	**186**	**184**	**370**	**136**	**142**	**278**	**122**	**117**	**239**	**115**	**102**	**217**	**143**	**109**	**252**	**123**	**98**	**221**
	Mean (SD)	71.7 (26.9)	68.7 (28.1)	70.2 (27.5)	60.3 (27.4)	60.8 (27.1)	60.5 (27.2)	58.1 (27.5)	59.8 (28.0)	58.9 (27.7)	60.4 (28.6)	60.8 (28.2)	60.6 (28.4)	61.7 (29.4)	58.3 (26.9)	60.2 (28.4)	56.9 (29.9)	56.3 (29.8)	56.6 (29.8)
**Patient EDE-Q**	**N**	**186**	**184**	**370**	**121**	**118**	**239**	**113**	**109**	**222**	**101**	**97**	**198**	**140**	**107**	**247**	**115**	**91**	**206**
	Mean (SD)	3.9 (1.4)	3.8 (1.3)	3.9 (1.4)	3.5 (1.4)	3.4 (1.4)	3.5 (1.4)	3.4 (1.5)	3.4 (1.4)	3.4 (1.5)	3.3 (1.5)	3.3 (1.6)	3.3 (1.5)	3.5 (1.6)	3.4 (1.4)	3.5 (1.5)	3.3 (1.4)	3.3 (1.5)	3.3 (1.5)
	Median (IQR)	4.4 (3.2-5.0)	4.3 (3.1-4.9)	4.3 (3.1-4.9)	3.7 (2.5-4.7)	3.6 (2.5-4.6)	3.6 (2.5-4.7)	3.5 (2.4-5.0)	3.5 (2.3-4.5)	3.5 (2.3-4.8)	3.4 (2.2-4.7)	3.4 (1.9-4.9)	3.4 (2.0-4.7)	3.7 (2.2-4.9)	3.4 (2.3-4.4)	3.6 (2.2-4.7)	3.5 (2.1-4.5)	3.4 (2.3-4.6)	3.5 (2.2-4.5)
**Patient reported BMI**	**N**	**179**	**179**	**358**	**131**	**132**	**263**	**109**	**108**	**217**	**102**	**94**	**196**	**132**	**98**	**230**	**112**	**87**	**199**
	Mean (SD)	15.9 (2.1)	15.9 (2.0)	15.9 (2.0)	17.4 (2.2)	16.9 (1.9)	17.1 (2.1)	17.3 (2.5)	17.0 (2.4)	17.2 (2.4)	17.5 (2.6)	16.9 (2.2)	17.2 (2.5)	17.4 (2.8)	16.9 (2.3)	17.2 (2.6)	17.6 (2.7)	17.3 (2.7)	17.5 (2.7)
**Patient WSAS**	**N**	**185**	**184**	**369**	**--**	**--**	**--**	**111**	**104**	**215**	**--**	**--**	**--**	**134**	**108**	**242**	**115**	**91**	**206**
	Mean (SD)	25.0 (9.3)	23.3 (9.2)	24.1 (9.3)	--	--	--	20.3 (9.9)	19.1 (9.9)	19.7 (9.9)	--	--	--	18.0 (12.0)	18.4 (10.8)	18.2 (11.5)	16.0 (10.8)	17.0 (11.8)	16.5 (11.3)
	Median (IQR)	26.0 (18.0-32.0)	24.0 (17.0-30.0)	25.0 (17.0-31.0)	--	--	--	20.0 (12.0-29.0)	18.0 (10.5-27.5)	19.0 (11.0-28.0)	--	--	--	17.0 (8.0-28.0)	17.0 (9.0-27.0)	17.0 (8.0-27.0)	14.0 (7.0-24.0)	15.0 (8.0-25.0)	14.0 (7.0-24.0)
	**N**	**185**	**184**	**369**	**121**	**118**	**239**	**113**	**109**	**222**	**--**	**--**	**--**	**134**	**107**	**241**	**114**	**91**	**205**

**Patient Motivation to change**	Mean (SD)	7.0 (2.8)	7.1 (2.7)	7.1 (2.8)	7.2 (2.8)	7.3 (2.4)	7.3 (2.6)	7.1 (2.7)	7.2 (2.5)	7.1 (2.6)	--	--	--	6.8 (2.9)	7.0 (2.6)	6.9 (2.8)	6.8 (2.7)	6.6 (3.0)	6.7 (2.8)
	Median (IQR)	8.0 (5.0-10.0)	8.0 (5.0-10.0)	8.0 (5.0-10.0)	8.0 (6.0-10.0)	8.0 (6.0-9.0)	8.0 (6.0-10.0)	8.0 (6.0-10.0)	8.0 (6.0-9.0)	8.0 (6.0-9.0)	--	--	--	8.0 (4.0-10.0)	7.0 (5.0-10.0)	8.0 (5.0-10.0)	7.5 (5.0-9.0)	7.0 (4.0-10.0)	7.0 (5.0-9.0)
	**N**	**185**	**184**	**369**	**121**	**118**	**239**	**113**	**109**	**222**	**--**	**--**	**--**	**134**	**108**	**242**	**114**	**91**	**205**
**Patient Ability to change**	Mean (SD)	3.9 (2.6)	4.2 (2.7)	4.0 (2.7)	3.9 (2.6)	4.2 (2.6)	4.1 (2.6)	4.1 (2.6)	4.0 (2.7)	4.1 (2.6)	--	--	--	3.8 (2.8)	3.9 (2.6)	3.8 (2.7)	4.1 (2.7)	3.9 (2.7)	4.0 (2.7)
	Median (IQR)	3.0 (2.0-6.0)	3.0 (2.0-6.0)	3.0 (2.0-6.0)	3.0 (2.0-6.0)	4.0 (2.0-6.0)	4.0 (2.0-6.0)	4.0 (2.0-6.0)	3.0 (2.0-6.0)	4.0 (2.0-6.0)	--	--	--	3.0 (2.0-6.0)	3.0 (2.0-6.0)	3.0 (2.0-6.0)	4.0 (2.0-6.0)	3.0 (2.0-6.0)	3.0 (2.0-6.0)
**Patient SDQ**	**N**	**185**	**182**	**367**	**--**	**--**	**--**	**--**	**--**	**--**	**--**	**--**	**--**	**107**	**83**	**190**	**--**	**--**	**--**
Total scale		19.6 (6.1)	18.8 (5.5)	19.2 (5.8)	--	--	--	--	--	--	--	--	--	15.9 (6.9)	17.2 (7.1)	16.5 (7.0)	--	--	--
Peer problems subscale	Mean (SD)	3.4 (2.0)	3.3 (1.9)	3.3 (2.0)	--	--	--	--	--	--	--	--	--	3.0 (2.0)	3.1 (2.0)	3.0 (2.0)	--	--	--
Prosocial subscale		6.5 (2.2)	6.6 (2.2)	6.6 (2.2)	--	--	--	--	--	--	--	--	--	6.8 (2.3)	6.7 (2.0)	6.8 (2.2)	--	--	--
**Patient CSRI**	**N**	**185**	**184**	**369**	**--**	**--**	**--**	**--**	**--**	**--**	**--**	**--**	**--**	**134**	**107**	**241**	**--**	**--**	**--**
In-patient hospital days during prior 3 months	Mean (SD)	51.0 (35.6)	52.8 (34.9)	51.9 (35.3)	--	--	--	--	--	--	--	--	--	14.4 (30.5)	14.9 (31.3)	14.7 (30.8)	--	--	--
	Median (IQR)	46.0 (23.0 – 81.0)	56.0 (28.0 – 82.0)	52.0 (25.0 – 82.0)	--	--	--	--	--	--	--	--	--	0.0 (0.0-1.0)	0.0 (0.0-3.0)	0.0 (0.0-1.0)	--	--	--
**ED-5D-5L**	**N**	**185**	**184**	**369**										**132**	**108**	**240**			

	Mean	0.47	0.52	0.49										0.54	0.58	0.56			
	(SD)	0.31	0.32	0.32										0.32	0.29	0.31			
	Median	0.41	0.62	0.52										0.62	0.69	0.69			
	(IQR)	(0.26-0.79)	(0.26 – 0.81)	(0.26-0.81)										(0.27 - 0.84)	(0.29 – 0.81)	(0.27 – 0.81)			
**Carer DASS-21**	**N**	**186**	**184**	**370**	**136**	**130**	**266**	**112**	**111**	**223**	**97**	**85**	**182**	**114**	**95**	**209**	**102**	**79**	**181**
	Mean	31.7	32.1	31.9	30.9	29.9	30.4	30.4	31.4	30.9	26.1	27.6	26.8	29.6	27.6	28.7	29.7	26.6	28.4
	(SD)	(24.9)	(23.8)	(24.3)	(22.6)	(20.6)	(21.6)	(24.5)	(23.8)	(24.1)	(20.2)	(20.1)	(20.1)	(22.7)	(18.7)	(20.9)	(22.5)	(18.9)	(21.0)
	Median (IQR)	26.0 (12.0-42.0)	26.0 (14.0-44.0)	26.0 (14.0-44.0)	29.0 (14.0-42.0)	26.0 (14.0-42.0)	27.0 (14.0-42.0)	24.0 (13.0-41.0)	26.0 (12.0-40.0)	24.0 (12.0-40.0)	20.0 (12.0-38.0)	24.0 (14.0-34.0)	22.0 (12.0-38.0)	25.0 (12.0-40.0)	24.0 (16.0-36.0)	24.0 (14.0-38.0)	27.0 (14.0-38.0)	22.0 (14.0-34.0)	26.0 (14.0-36.0)
**Carer CASK**	**N**	**185**	**182**	**367**	**--**	**--**	**--**	**98**	**97**	**195**	**--**	**--**	**--**	**108**	**86**	**194**	**96**	**74**	**170**
	Mean	156.4	156.5	156.5	--	--	--	155.0	154.9	155.0	--	--	--	164.8	152.6	159.4	161.0	170.2	165.0
	(SD)	(41.3)	(40.2)	(40.7)				(49.8)	(44.6)	(47.2)				(50.3)	(52.3)	(51.5)	(54.0)	(50.7)	(52.6)

Note. For continuous measures with skewed distributions, both the mean (SD) and median (IQR) are provided.

Supplement 7.

Summary of online forum sessions attended by patient and carer participants throughout the intervention period by group type

Group type	Patients N (%)	Carers N (%)	Patient/carer dyad (N (%)
Total groups	82 (45)	82 (45)	62 (34)
Patient-only	76 (41)	-	-
Carer-only	-	72 (39)	-
Patient/carer joint	57 (31)	60 (33)	40 (22)

Note. Data presented based on participation in minimum of one online group of each type. Total groups indicates the sum of patient-only, or carer-only groups, and joint groups.

Supplement 8.

Cost-effectiveness plane – societal perspective

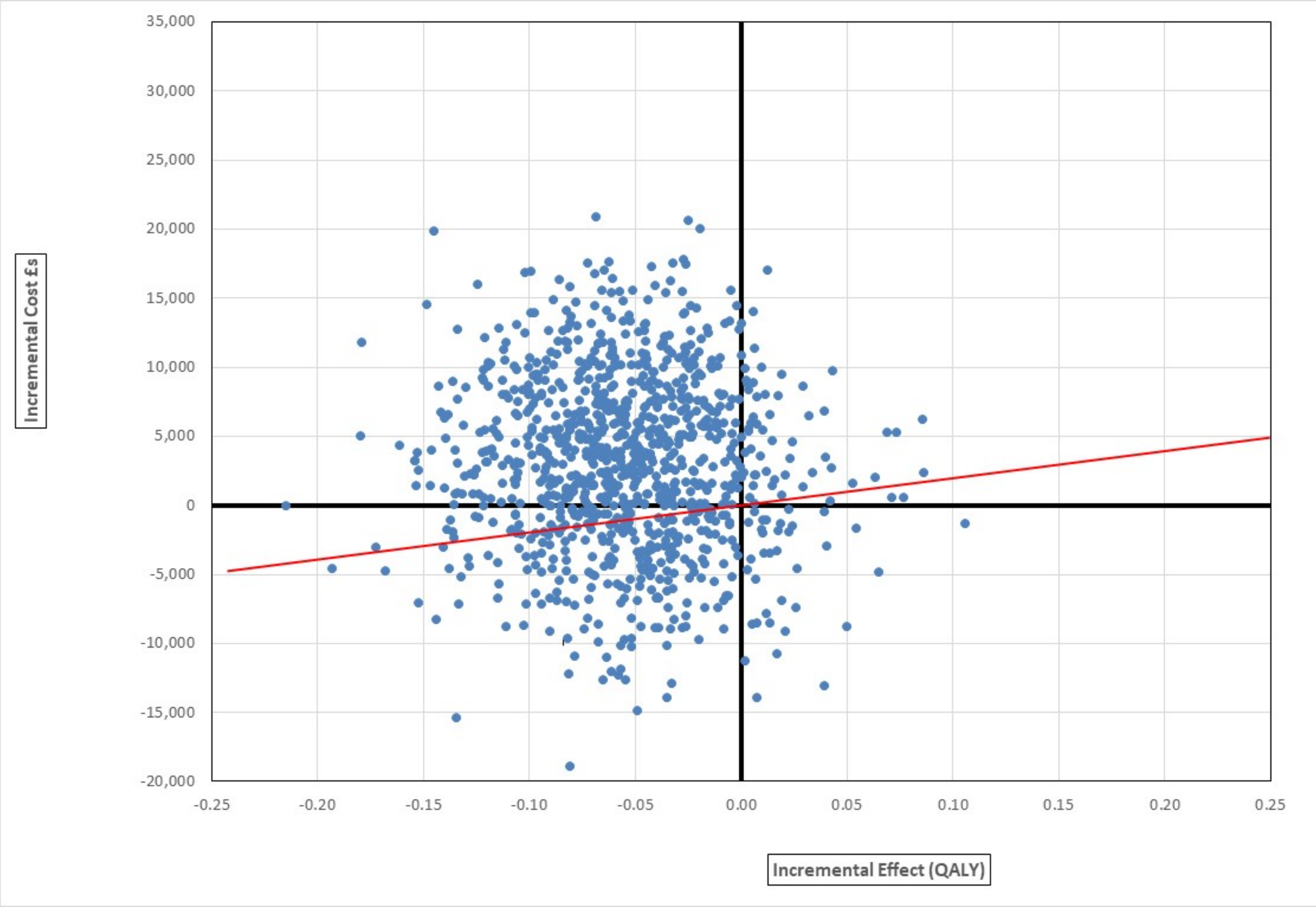

Supplement 9.

Mean Difference in Service Utilisation Per Participant at 12 Month Follow Up (previous 3 months) (Imputed dataset)

Type of Contact	ECHOMANTRA /TAU N=184	TAU N=185	Mean Difference (BCa 95% CI)*	*P*
Health Service Utilisation, *M (SD)*				
Eating disorder inpatient stay (nights)	15.68 (26.32)	14.23 (26.00)	1.45 (-4.09, 6.89)	0.588
General inpatient psychiatric stay (nights)	1.92 (5.43)	1.59 (7.17)	0.33 (-1.09, 1.58)	0.671
Other inpatient stay (nights)	1.96 (5.14)	1.12 (3.25)	0.84 (-0.35, 1.71)	0.074
Eating Disorders Day Patient Visits (days)	1.30 (3.60)	2.18 (8.47)	-0.89 (-2.30, 0.33)	0.236
Hospital Outpatient Visits (visits)	4.08 (5.97)	5.25 (8.59)	-1.16 (-2.77, 0.33)	0.129
A&E/Minor Injury Clinic (visits	0.47 (1.05)	0.41 (0.86)	0.07 (-0.12, 0.26)	0.551
GP (contacts)	1.69 (2.40)	2.23 (3.28)	-0.55 (-1.13, 0.02)	0.066
GP Practice Nurse (contacts)	7.20 (13.89)	4.06 (10.03)	3.15 (0.80, 5.80)	**0.010**
Dietician (contacts)	5.19 (9.62)	3.57 (8.19)	1.62 (-0.36, 3.52)	0.084
Dentist (contacts)	1.00 (2.31)	0.52 (1.01)	0.48 (0.01, 0.18)	**0.013**
Other services (contacts)	4.83 (8.81)	8.1 (19.49)	-3.27 (-6.51, -0.49)	**0.05**
All inpatient stays (nights)	19.57 (27.17)	16.94 (27.39)	2.62 (-3.06, 8.74)	0.348
All A&E / daypatient/ outpatient (contacts)¥	5.85 (7.22)	7.83 (12.64)	-1.98 (-4.28, 0.04)	0.086
All community service (contacts)	19.91 (21.82)	18.48 (26.10)	1.43 (-3.85, 6.47)	0.572
Productivity Loss (hours)	64.12 (120.40)	72.03 (129.20)	-7.90 (-32.01, 19.24)	0.551

*Bias corrected accelerated bootstraps
¥ each daypatient stay assumed to be one contact

Supplement 10.

Comparison of previous 3-month costs at 12-month follow up and QALYs gained ECHOMANTRA group who did or did not complete at least 4 online group sessions (Imputed dataset) 2022 £s

Outcome at 12 months	At least 4 online group sessions completed N=36	Less than 4 online group sessions completed N=148	Mean Difference (BCa 95% CI)*	*P*
	Mean (SD)			
All inpatient costs	8,163 (18,507)	12,781 (16,839)	-4,617 (10,466, 1,564)	0.19
Total health system costs	9,791 (19,066)	15,425 (17,181)	-1,912 (-17,987, 12,479)	0.80
Productivity losses	950 (1,693)	2,178 (3,131)	-1,228 (-1,933, -478)	**0.006**
Out of pocket expenses	125 (183)	273 (375)	-149 (-235, -72)	**0.002**
QALYs gained	0.618 (0.257)	0.501 (0.305)	0.108 (0.007, 0.196)	**0.045**
QALY change	0.035 (0.318)	0.001 (0.318)	0.034 (-0.080, 0.148)	0.19

Supplement 11.

Patient adverse events by trial arm and overall

	TAU	TAU+ECHOMANTRA	Overall
Is the event serious?<br>N (%)			
No	3 (3.2)	2 (2.2)	5 (2.7)
Yes	91 (96.8)	87 (97.8)	178 (97.3)
Adverse event type<br>Events (people)			
Cardiovascular	0 (0)	2 (2)	2 (2)
Respiratory	1 (1)	1 (1)	2 (2)
Gastro-intestinal	2 (2)	0 (0)	2 (2)
Genito-urinary/renal	0 (0)	1 (1)	1 (1)
Psychological	89 (67)	84 (61)	173 (128)
Immunological	2 (2)	0 (0)	2 (2)
Relationship of adverse event to study participation<br>N (%)			
Definitely related	0 (0.0)	1 (1.1)	1 (0.5)

Not related	94 (100.0)	88 (98.9)	182 (99.5)

* These were associated to anxiety and upset triggered by completing the self-report questionnaires.

Supplement 12.

Carer adverse events, by trial arm and overall

	TAU	ECHOMANTRA	Overall
Is the event serious?<br>N (%)			
No	2 (100.0)	3 (100.0)	5 (100.0)
Adverse event type – Events (people)			
Respiratory	0 (0)	2 (2)	2 (2)
Psychological	2 (2)	1 (1)	3 (3)
Intensity<br>N (%)			
Mild	2 (100.0)	2 (66.7)	4 (80.0)
Moderate	0 (0.0)	1 (33.3)	1 (20.0)
Relationship to study participation<br>N (%)			
Definitely related*	2 (100.0)	0 (0.0)	2 (40.0)
Not related	0 (0.0)	3 (100.0)	3 (60.0)

* These were associated to anxiety and upset triggered by completing the self-report questionnaires.

References

Allison, C., Auyeung, B., & Baron-Cohen, S. (2012). Toward brief "red flags" for autism screening: the short autism spectrum quotient and the short quantitative checklist in 1,000 cases and 3,000 controls. *Journal of the American Academy of Child & Adolescent Psychiatry, 51*(2), 202-212. e207.

Beecham, J., & Knapp, M. (1992). Costing psychiatric interventions. In G. Thornicroft, C. Brewin, & J. Wing (Eds.), *Measuring Mental Health Needs* (pp. 163-183). London, England: Gaskell.

Beecham, J., & Knapp, M. (2015). Client Service Receipt Inventory. In.

Fairburn, C. G., & Beglin, S. J. (1994). Assessment of eating disorders: interview or self-report questionnaire? *Int J Eat Disord, 16*(4), 363-370. Retrieved from https://www.ncbi.nlm.nih.gov/pubmed/7866415

Foa, E. B., Huppert, J. D., Leiberg, S., Langner, R., Kichic, R., Hajcak, G., & Salkovskis, P. M. (2002). The Obsessive-Compulsive Inventory: development and validation of a short version. *Psychological Assessment, 14*(4), 485.

Goodman, R. (1997). The Strengths and Difficulties Questionnaire: a research note. *Journal of Child Psychology and Psychiatry, 38*(5), 581-586.

Herdman, M., Gudex, C., Lloyd, A., Janssen, M., Kind, P., Parkin, D., . . . Badia, X. (2011). Development and preliminary testing of the new five-level version of EQ-5D (EQ-5D-5L). *Quality of life research, 20*(10), 1727-1736.

Hibbs, R., Rhind, C., Salerno, L., Lo Coco, G., Goddard, E., Schmidt, U., . . . Macdonald, P. (2015). Development and validation of a scale to measure caregiver skills in eating disorders. *International Journal of Eating Disorders, 48*(3), 290-297.

Kind, P., Dolan, P., Gudex, C., & Williams, A. (1998). Variations in population health status: results from a United Kingdom national questionnaire survey. *bmj, 316*(7133), 736-741. doi:10.1136/bmj.316.7133.736

Lovibond, S., & Lovibond, P. (1995). Depression anxiety stress scale-21 (DASS-21). *Sydney: School of Psychology, University of New South Wales, 10.*

Mundt, J. C., Marks, I. M., Shear, M. K., & Greist, J. M. (2002). The Work and Social Adjustment Scale: a simple measure of impairment in functioning. *The British Journal of Psychiatry, 180*(5), 461-464.

National Institute for Health and Care Excellence. (2019). Position statement on use of the EQ-5D-5L value set for England (updated October 2019). https://www.nice.org.uk/about/what-we-do/our-programmes/nice-guidance/technology-appraisal-guidance/eq-5d-5l (Accessed 26 July 2023).
